# Bacterial Exposure Mediates Developmental Plasticity and Resistance to Lethal *Vibrio lentus* Infection in Purple Sea Urchin *(Strongylocentrotus purpuratus)* Larvae

**DOI:** 10.3389/fimmu.2019.03014

**Published:** 2020-01-14

**Authors:** Nicholas W. Schuh, Tyler J. Carrier, Catherine S. Schrankel, Adam M. Reitzel, Andreas Heyland, Jonathan P. Rast

**Affiliations:** ^1^Department of Medical Biophysics, University of Toronto, Toronto, ON, Canada; ^2^Department of Biological Sciences, Sunnybrook Research Institute, Toronto, ON, Canada; ^3^Department of Integrative Biology, University of Guelph, Guelph, ON, Canada; ^4^Department of Biological Sciences, University of North Carolina at Charlotte, Charlotte, NC, United States; ^5^Department of Immunology, University of Toronto, Toronto, ON, Canada; ^6^Marine Biology Research Division, Scripps Institute of Oceanography, University of California, San Diego, San Diego, CA, United States; ^7^Department of Pathology and Laboratory Medicine, Emory University School of Medicine, Atlanta, GA, United States; ^8^Emory Vaccine Center, Emory University, Atlanta, GA, United States

**Keywords:** sea urchin, larva, development, immunity, plasticity, *Vibrio*, microbiome, bacteria

## Abstract

Exposure to and colonization by bacteria during development have wide-ranging beneficial effects on animal biology but can also inhibit growth or cause disease. The immune system is the prime mediator of these microbial interactions and is itself shaped by them. Studies using diverse animal taxa have begun to elucidate the mechanisms underlying the acquisition and transmission of bacterial symbionts and their interactions with developing immune systems. Moreover, the contexts of these associations are often confounded by stark differences between “wild type” microbiota and the bacterial communities associated with animals raised in conventional or germ-free laboratories. In this study, we investigate the spatio-temporal kinetics of bacterial colonization and associated effects on growth and immune function in larvae of the purple sea urchin (*Strongylocentrotus purpuratus*) as a model for host-microbe interactions and immune system development. We also compare the host-associated microbiota of developing embryos and larvae raised in natural seawater or exposed to adult-associated bacteria in the laboratory. Bacteria associated with zygotes, embryos, and early larvae are detectable with 16S amplicon sequencing, but 16S-FISH indicates that the vast majority of larval bacterial load is acquired after feeding begins and is localized to the gut lumen. The bacterial communities of laboratory-cultured embryos are significantly less diverse than the natural microbiota but recapitulate its major components (Alphaproteobacteria, Gammaproteobacteria, and Bacteroidetes), suggesting that biologically relevant host-microbe interactions can be studied in the laboratory. We also demonstrate that bacterial exposure in early development induces changes in morphology and in the immune system. In the absence of bacteria, larvae grow larger at the 4-arm stage. Additionally, bacteria-exposed larvae are significantly more resistant to lethal infection with the larva-associated pathogen *Vibrio lentus* suggesting that early exposure to high levels of microbes, as would be expected in natural conditions, affects the immune state in later larvae. These results expand our knowledge of microbial influences on early sea urchin development and establish a model in which to study the interactions between the developing larval immune system and the acquisition of larval microbiota.

## Introduction

Bacterial microbiota influence diverse aspects of metazoan development and physiology (1–4). Notable examples include colonization and fluorescence of *Aliivibrio fischeri* in the light organ of the Hawaiian bobtail squid (*Euprymna scolopes*) (5, 6), participation in digestion and metabolism in phyla from poriferans to chordates (7, 8), and numerous studies linking bacterial dysbiosis to immune dysfunction and disease in diverse animal taxa [e.g., corals (9), sponges (10), honeybees (11), and mammals (4, 12–15)]. However, much remains to be understood regarding the acquisition of bacterial microbiota and their interactions with the immune systems of developing hosts—for instance, the roles of microbial exposure on immune development and function, potential trade-offs between growth and immunity, and whether natural microbiota can be meaningfully reproduced in the laboratory. In this study, we examine the effects of bacterial colonization on the early development of the purple sea urchin (*Strongylocentrotus purpuratus*), an important developmental model, and show that such exposure is associated with both morphological changes and resistance to challenge with a pathogenic *Vibrio*.

Sea urchins are marine deuterostome invertebrates in the phylum Echinodermata, which, with the hemichordates, form the sister group to the chordates. Echinoderms have been important research organisms in cell and developmental biology from the work of the nineteenth century embryologists to modern day (16–19). Ilya Metchnikoff first described cellular immunity in echinoderm larvae in the late 1800s (20), and recently, researchers have returned to these organisms with modern genomic, experimental, and imaging techniques, describing the specialized immune cell types of echinoderm larvae and their functions in response to diverse challenges [e.g., (21–24); reviewed in (17, 19, 25)]. In parallel, embryonic, larval, and adult echinoderm bacterial microbiomes have been characterized in several species [e.g., (22, 26–31); reviewed in (19)]. These studies focused on bacteria acquired in natural seawater or in the wild, but major questions that remain are to what degree artificial seawater-raised laboratory animals recapitulate the microbiota of their wild counterparts (32, 33) and how these differences may affect the developing immune system and the emergent bacterial microbiota.

Phenotypic plasticity, a single genome giving rise to multiple phenotypes under different environmental conditions, has been widely studied in sea urchin and other echinoderm larvae [Reviewed in (34, 35)]. Planktotrophic larvae tend to undergo a plastic response to fluctuating food availability. Specifically, under low-food conditions, the lengths of the larval arms, used for feeding, are increased relative to larval body length, while overall gut volume is reduced (35–39). Recently, Carrier and Reitzel (28) correlated feeding-induced plasticity with structural changes in the associated bacterial microbiota of three confamilial sea urchins (*S. purpuratus, S. droebachiensis*, and *Mesocentrotus franciscanus*), underscoring a role for microbes in larval development (19, 31). However, the degree to which bacteria drive phenotypic plasticity irrespective of feeding, and whether they participate in other aspects of larval development, remain unclear.

*Vibrio*s are a widespread genus of aquatic Gammaproteobacteria that are pathogenic in a diverse array of organisms, including larval sea urchins (e.g., *V. diazotrophicus, V. cyclitrophicus*, and *V. splendidus*) (22), adult sea urchins (e.g., *V. diazotrophicus, V. anguillarum*) (40), and humans (e.g., *V. cholerae, V. vulnificus*, and *V. parahemolyticus*) (41). In *S. purpuratus* larvae, exposure to *V. diazotrophicus* induces numerous changes in immune cell behavior: the gut epithelium thickens, occluding the lumen, and specialized immunocytes become active—most notably pigment cells, which round up, migrate from the ectoderm into the blastocoel and to the gut surface, and form associations with other immune cells, including globular and filopodial cells (19, 22). In later infection, *V. diazotrophicus* invades the blastocoel and is phagocytosed by a subclass of phagocytic cells (22). *V. diazotrophicus* was isolated from the guts of adult green sea urchins (*Strongylocentrotus droebachiensis*) in the northern Atlantic (42). For this report, we isolated bacterial strains directly from *S. purpuratus* larvae in order to study more intimate host-microbe associations (22).

Although originally thought to be environmental or commensal organisms with little pathogenic significance (43), *V. splendidus* and its close relatives (henceforth *Splendidus* clade *Vibrio*s) have been shown to cause disease in many aquatic organisms, both wild and farmed [e.g., bivalves (44–47), octopuses (48), turbot (49, 50), and eels (51)]. Seven *Splendidus* clade strains were previously isolated from 4-arm *S. purpuratus* larvae, two of which were tested in large scale exposures (22). One strain, identified as *V. cyclitrophicus*, induced robust immune responses, while the other, a *V. splendidus* strain, was lethal (22). A third strain, identified as *V. lentus* in this study, induces rapid (<30 min) multicellular immune responses upon blastocoelar injection, and is also lethal to larvae (19). The mechanisms through which these *Vibrio* strains cause disease in larval and adult sea urchins are unknown.

Here, we investigate the spatiotemporal kinetics of bacterial colonization and demonstrate that the vast majority of larval bacterial load is acquired at feeding stage and localized to the gut lumen. We also compare the microbiomes of natural seawater- and laboratory-raised *S. purpuratus* larvae in early development and show that larvae exposed to environmentally relevant bacteria and other microbes in the laboratory exhibit developmental plasticity that is distinct from feeding-induced plasticity. Finally, we characterize infection with *V. lentus*, a larva-associated pathogen, and utilize this bacterium to assay the effects of early exposure to microbiota on the developing larval immune system. Bacteria-exposed larvae develop more slowly and are significantly smaller than larvae raised under conditions of low bacterial load, but are substantially more resistant to lethal *Vibrio* infection, indicating a potential role for immune priming by early exposure to microbiota. These results expand the scope of bacterial involvement in phenotypic plasticity and highlight a role for microbiota in the normal development of the larval immune system.

## Materials and Methods

### Animals

Laboratory adult purple sea urchins were obtained from Point Loma Marine Invertebrate Lab (Lakeside, California, USA), and maintained in recirculating 34 ppt salinity artificial seawater (FSW; Instant Ocean®) at 15°C, and fed Giant kelp (*Macrocystis pyrifera*) collected from their local environment or dried commercial *Kombu* cuttings twice weekly. For natural microbiota experiments, adult animals were collected and maintained as described in Carrier and Reitzel (31). Briefly, animals were gathered during low tide at Slip Point, Clallam Bay (Washington, USA) and transferred directly to Friday Harbor Laboratories where they were suspended in sub-tidal cages off the dock and fed *Nereocystis* spp. (sugar kelp) *ad libitum*. Animal protocols were approved by the Sunnybrook Research Institute Animal Care Committee.

### Sterilized and Inoculated Media

Sterilized artificial seawater (P/S) was prepared from FSW (see above) by 0.2 μm vacuum filtration (EMD Millipore) and the addition of 100 U/mL penicillin and 100 μg/mL streptomycin (ThermoFisher Scientific). Adult-exposed inoculated seawater (AEW) was prepared from FSW by addition of 20% (v/v) water from the aquaria used to house our adult animals, then filtered through 40 μm mesh to remove debris and small organisms. Medium pH was measured using a FE20-Basic FiveEasy™ benchtop pH meter (Mettler Toledo). Culturable bacterial load was measured by spreading 25 μL of each medium on 10 cm Zobell marine broth agar (52) (BD Difco) plates, incubating in a humidified chamber for 3 days at 15°C, and counting the resulting colony-forming units (CFU). Counting was performed with the naked eye while plates were illuminated from beneath.

### Larval Cultures

Spawning was induced by manual agitation or injection of 0.5–1.5 mL 0.5 M KCl, depending on animal size. Eggs were filtered through 80 μm mesh to remove debris and settled through three FSW washes prior to fertilization with FSW-diluted sperm (~1/250,000). Following three additional washes, cultures were established at 20,000 zygotes/L in FSW at 15°C and stirred at 30 rpm. Cultures were diluted to ~2–5 larvae/mL at 4–5 days post fertilization (dpf) prior to feeding with *Rhodomonas lens* (5,000 cells/mL). Feeding larvae were transferred into clean FSW with fresh *R. lens* every 2–3 days.

### Larval Bacterial Load Cultures, 16S Sequencing Cultures, and Environmental Sample Collection

Bacterial load experiments were performed as above with the following modifications: eggs and sperm were spawned from same mate pair into P/S, FSW, or AEW. Fertilization, zygote washes, and embryo/larva water changes were performed in the appropriate medium. A separate *Rhodomonas* culture maintained in 0.2 μm-filtered f/2 medium (Sigma-Aldrich) with 100 U/mL penicillin and 100 μg/mL streptomycin was used to feed the low bacterial load (P/S) cohort. This treatment did not noticeably affect algal quality, and cultures were washed and resuspended in FSW prior to use.

Laboratory (AEW)- and natural seawater (NSW)-exposed larvae raised for comparison by 16S rRNA sequencing were cultured as above but collected somewhat differently, reflecting standard practices used by inland and field-based laboratories in previous studies [e.g., (22) vs. (28, 38)]. Laboratory-reared larvae were raised in AEW but washed to remove unassociated microorganisms and flocculent debris prior to sample processing (see below). This was performed by manually transferring larvae three times in small volumes through dishes of 0.2 μm-filtered FSW (22). These animals were then starved at ~2–5 larvae/mL in 0.2 μm-filtered FSW overnight prior to sample processing to minimize the contribution from microorganisms that were only transiently present in the gut lumen. NSW-raised larvae were collected directly by centrifugation and seawater removal (28). In all cases, treatment-matched *Rhodomonas* samples were spun down at 800 *g* for 3 min, washed in FSW, and similarly processed. All sea urchin and *Rhodomonas* samples were stored at −20°C in 50 μL of saltwater isotonic TE (1 mM EDTA, 10 mM Tris HCl pH 7.5, 0.5 M NaCl). In parallel, particles (i.e., prokaryotes, fungi, and other debris) from matched AEW (laboratory) or natural seawater samples (~250–500 mL) were retained on 0.2 μm syringe filters (Corning) and sealed prior to freezing and processing. All 16S rRNA sequencing samples were collected randomly from 3–5 biological replicates (independent broods), processed, and sequenced in parallel (see below).

### 16S Fluorescent *in situ* Hybridization

We measured the spatial and temporal kinetics of bacterial colonization using fluorescent *in situ* hybridization (FISH) with the domain Bacteria 16S rRNA probe EUB338 (53) 5′-conjugated to Cy5 (Integrated DNA Technologies) using a protocol modified from Stahl and Amann (54) and described in Ho et al. (22). Briefly, embryos and larvae were fixed overnight in 4% paraformaldehyde then hybridized overnight with 1 ng/ μL probe at 46°C. Pre-hybridization, hybridization, and washes were all performed in 0.2 μm-filtered 900 mM NaCl, 20 mM Tris HCl (pH 7.5), 0.01% SDS (v/v). Specimens were counterstained with 2 nM Hoechst for 15–30 min at room temperature, mounted in TBST (0.2 M Tris pH 7.5, 0.15 M NaCl, 0.1% (v/v) Tween20) with 1–2 drops of an antifadent (Citfifluor AF1, Electron Microscopy Sciences), and imaged within 2–3 days of staining. Larvae-associated bacteria were counted manually from z-stack images using the cell counter plugin in ImageJ. Each stained bacterium was only counted in the z slice at which it appeared in maximum focus, ensuring that cells were not counted twice.

### 16S Community Profiling

Total DNA was extracted from embryonic and larval samples using the GeneJet Genomic DNA Purification Kit (Thermo Scientific). DNA from environmental AEW and NSW samples were extracted using the FastDNA Spin Kit for Soil (MP Biomedical). DNA was then quantified using the NanoDrop 2000 UV-Vis Spectrophotometer (Thermo Scientific) and diluted to 5 ng/μL using RNase/DNase-free water. Bacterial sequences were amplified using universal primers for the V3/V4 regions of the 16S rRNA gene (F: 5′ CTACGGGNGGCWGCAG, R: 5′ GACTACHVGGGTATCTAATCC) (55) in 25 μL reaction volumes with Kapa Ready Mix (Sigma Aldrich) with an initial melting stage at 95°C for 3 min, 25 cycles of 95°C for 30 s, 55°C for 30 s, and 72°C for 30 s, followed by a final elongation stage at 72°C for 5 min. Products were purified using the Axygen AxyPrep Mag PCR Clean-up Kit (Axygen Scientific, New York, USA), indexed via PCR (as above, for 8 cycles) using the Nextera XT Index Kit V2 (Illumina, California, USA), and then purified again. At each of these three steps, fluorometric quantitation was performed using a Qubit (Life Technologies, California, USA) and libraries were validated using a Bioanalyzer High Sensitivity DNA chip (Agilent Technologies, California, USA). Illumina MiSeq sequencing (v3, 2 × 300 bp paired-end reads) was performed at the University of North Carolina at Charlotte.

Forward and reverse sequences were paired and trimmed using PEAR (56) and Trimmomatic (57), respectively, converted from fastq to fasta using a custom script, and chimeric sequences were detected using USEARCH (58) and removed using filter_fasta.py prior to the analysis of bacterial 16S rRNA sequences. Using QIIME 1.9.1 [or Quantitative Insights Into Microbial Ecology; (59)], bacterial 16S rRNA sequences were analyzed and grouped into operational taxonomic units (OTUs) based on a minimum 97% similarity. The biom table generated by the pick_open_reference_otus.py script was filtered of OTUs with 10 or fewer reads as well as sequences matching the chloroplasts of cryptophytes [i.e., *R. lens*; (28)].

Using the filtered biom table and “biom summarize-table” to count total sequences per sample, the rarefaction depth of 18,350 reads was determined and applied to all subsequent analyses (Supplementary Figure 3). Alpha diversity (i.e., Faith's phylogenetic distance and observed OTUs) was calculated using alpha_diversity.py and compared statistically using one-way ANOVAs in JMP. Beta diversity was calculated using both unweighted and weighted UniFrac (60) as part of jackknifed_beta_diversity.py. These values were compared using principal coordinate analyses (PCoA), recreated with make_2d_plots.py, and stylized for presentation in Adobe Illustrator CS6. Community similarity between NSW- and AEW-raised embryos and larvae were compared statistically using an analysis of similarity (ANOSIM) using compare_categories.py. The shared OTUs between urchin populations were determined using compute_core_microbiome.py and shared_phylotypes.py. Average bacterial community for each developmental stage and culturing condition was generated using summarize_taxa_through_plots.py, visualized with Prism 7 (GraphPad Software), and stylized for presentation in Adobe Illustrator CS6. A step-by-step listing of QIIME scripts used to convert raw reads to OTUs for visualization of the data is located in Supplementary Note 1. All AEW- and NSW-derived samples were processed, amplified, sequenced, and analyzed in parallel. The raw sequence reads as part of this dataset are available on Dryad at https://doi.org/10.5061/dryad.qv9s4mw9v.

### DIC Microscopy, Fluorescence Imaging, and Counting Larva-Associated Bacteria

All imaging was performed on a Zeiss Axioplan or Observer Z.1 microscope with Zeiss HrM or MrC5 cameras (Zeiss). Z-stack slices were imaged at 1.0–2.5 μm thickness. Image processing and analysis were performed using Zeiss Axiovision or ImageJ software (61). EUB338-Cy5-stained bacteria were counted manually from z-stack images using the “cell counter” plugin in ImageJ. Each stained bacterium was only counted in the z slice at which it appeared in maximum focus, ensuring that cells were not counted twice.

### Embryonic and Larval Morphometrics

The lengths of larval bodies and the various skeletal rods were measured in 3 dimensions from z-stack images using ImageJ. Midgut (also referred to in the literature as the larval stomach) area was measured when its largest cross section was in maximum focus. Individual comparisons between treatments were analyzed by 2-way ANOVA with post-test Bonferroni corrections. Larval morphology was also analyzed between treatments by extracting two principle components that together explain more than 50% of the variance in the dataset. We then compared these PCAs to the original morphometrics data to find out which PCA is best explained by which morphometric measurement and used these values in an ANOVA.

### *Vibrio lentus* Isolation and Identification

Larvae-associated bacteria were isolated and cultured using a method modified from Ho et al. (22). As described above, larvae were experimentally colonized in FSW inoculated with 20% 40 μm-filtered adult-exposed aquarium water (AEW), fed from 5 dpf, and raised to 10 dpf. Larvae were then starved by transfer to an equivalent volume of 0.2 μm-filtered FSW and incubated overnight to shed transiently associated gut microbes, washed by transfer through three sequential dishes of 0.2 μm-filtered FSW (~15 mL/wash), pestle-macerated in 1.5 mL microtubes, and plated on marine broth agar (52). These plates were incubated for 2–3 days at 15°C in a humidified chamber. Individual bacterial colonies were cultured at 15°C, 250 rpm in marine broth until turbid, used to prepare glycerol stocks for storage at −80°C, and pelleted by centrifugation prior to genomic DNA extraction with TRIzol (Invitrogen).

Multilocus sequence analysis was performed using 16S, *rpoD*, and *toxR* sequences amplified by PCR using primers listed in Supplementary Table 3 (62, 63). 16S amplicons were ligated into pCR4-TOPO vectors (Invitrogen), transfected into One Shot™ TOP10 competent *E. coli* (Invitrogen), and sequenced with T3 and T7 primers; *rpoD* and *toxR* sequences were amplified directly from genomic DNA. 16S sequences were amplified in 20 μL reaction volumes with 1.5 mM MgCl_2_, 0.2 mM dNTPs, 0.5 μM forward and reverse primers, and 0.025 U/uL Taq polymerase for an initial melting stage of 95°C for 30 s, 25 cycles of 95°C for 30 s, 50°C for 30 s, and 72°C for 30 s, followed by a final elongation stage at 72°C for 7 min. *rpoD* and *toxR* were amplified in 50 μL reaction volumes with 0.8 μM forward and reverse primers, 1.5 mM MgCl2, 0.2 mM dNTPs, 100–250 ng template DNA, and 0.025 U/μL Taq polymerase (ThermoFisher) following the thermal cycling protocol described in Pascual et al. (63): An initial 5 min melting stage at 95°C; 3 cycles of 95°C for 1 min, 55°C for 2 min 15 s, and 72°C for 1 min 15 s; 30 cycles of 95°C for 35 s, 55°C for 1 min 15 s, and 72°C for 1 min 15 s; and a final elongation stage at 72°C for 10 min. Amplicons were confirmed as single bands by 1.2% agarose gel electrophoresis and purified using Qiagen beads (Venlo, Netherlands) prior to sequencing at The Centre for Applied Genomics (Toronto, Canada), then manually inspected and trimmed using FinchTV (Geospiza) and BioEdit (64) software and referenced to the Ribosomal Data Project (65) and NCBI BLAST databases.

### *Vibrio lentus* Exposures

*Vibrio lentus* used for larval exposure were transferred from frozen glycerol stocks into 2 mL marine broth starter cultures at 15°C, 250 rpm and subcultured as necessary. Division was monitored by measuring absorbance at 600 nm and cultures were confirmed to be in an active growth phase prior to harvesting for larval exposure. Cells were counted in a Petroff-Hauser counting chamber at 400x magnification, washed and resuspended in FSW by centrifugation at ~3,500–5,000 *g* for 10–15 min, depending on volume, then added to larval cultures. Larval mortality was determined by observation at 100–200x magnification. Larvae with small amounts of adherent bacteria or ectodermal cell blebbing were considered alive, while lysed or skeletonized larvae were scored as dead.

### Experimental Design and Statistical Analysis

#### Differential Bacterial Load Larval Culture Media

Bacterial load was measured in P/S, FSW, and AEW as described in 10 independent experiments and analyzed by Kruskal-Wallis test followed by *post-hoc t*-testing (either 1-sample vs. hypothetical mean zero for FSW/AEW vs. P/S or 2-sample 2-tailed for FSW vs. AEW). Potential differences in variance were analyzed with Bartlett's test. Medium pH was measured in two independent experiments (Supplementary Figure 1).

#### 16S FISH

The images presented in Figure 2 and Supplementary Figure 2 are representative of >20 16S staining experiments performed on different developmental stages in independent broods over several years. The images in Figure 2A and Supplementary Figure 2E were taken of matched P/S, FSW, and AEW embryos and larvae from one brood. Supplementary Figure 2F quantifies EUB338-Cy5^+^ bacterial load in P/S, FSW, and AEW eggs, embryos, and 4 dpf larvae from this same brood (6–31 animals/group/time point, mean = 18 animals/group/time point), analyzed by 2-way ANOVA and *post-hoc* Bonferroni testing (comparison between each pair of groups, corrected for multiple testing). The images in Figure 2B are representative of NSW larvae from one brood (*n* = 19 animals). The images in Supplementary Figure 2A and quantified in Supplementary Figure 2B represent 24–36 eggs/ treatment pooled from 3 independent broods (indicated in figure) with stained bacterial load tested by 1-way ANOVA (which was not significant). The zygote images in Supplementary Figure 2C and quantified in Supplementary Figure 2D represent 10 zygotes/treatment from one brood, tested by 2-tailed *t* test (not significant).

#### Larval Morphometrics

The sizes of various developmental stages and larval structures in P/S, FSW, and AEW culture conditions were observed in several independent broods. Figure 4 illustrates the size of 1 dpf embryos (18–20 embryos/group) and 6, 8, and 11 dpf larvae (~10 larvae/group/time point) from one representative matched brood. Size differences between embryos were tested by 2-tailed *t* test. Differences between the sizes of larval structures were tested by 2-way ANOVA with *post-hoc* Bonferroni test (comparison between each pair of treatments at each time point, corrected for multiple testing). The reproduction of this effect in a second independent brood is illustrated in Supplementary Figure 8.

#### Immune Cell Behavior During *V. lentus* Exposure

Pigment cell rounding, pigment cell migration, midgut (gastric) epithelial hypertrophy, and survival were measured between larvae pooled from 1 to 5 independent broods at different doses (10^5^, 10^6^, and 10^7^ *V. lentus* cells/mL) at 6 and 24 h post-exposure. Homogeneity of variances was analyzed with Bartlett's tests. As some of these tests were significant, differences between groups were compared by Kruskal-Wallis and *post-hoc* Dunn's tests between each experimental group and the unexposed control.

#### Resistance to *V. lentus* Infection

AEW and P/S larvae were raised to 10 dpf and exposed to 10^7^ *V. lentus*/mL for 24 h in two independent experiments. Approximately nine larvae/treatment/experiment were randomly selected and scored for survival as described above.

**Figure 1:**
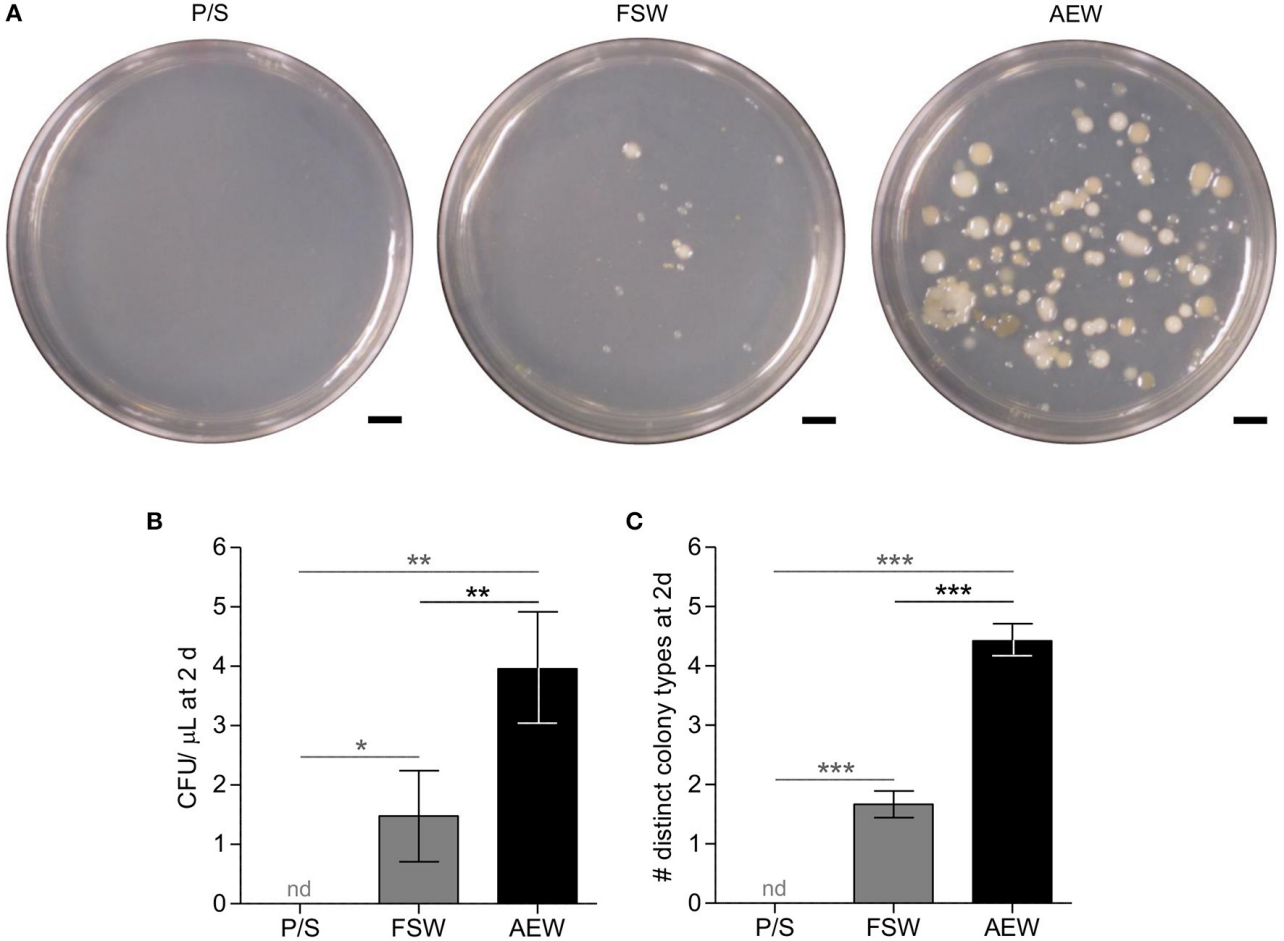
Filtration/antibiotic treatment and addition of adult-exposed water modify bacterial load and diversity in culture seawater. 0.22 μm-filtered FSW with 100 U/mL penicillin/and 100 μg/mL streptomycin (P/S), normal FSW, and FSW with 20% 40 μm-filtered adult-exposed tank water (AEW) were plated on marine broth agar and incubated at 14–15°C. **(A)** plates photographed after 7 days. **(B,C)** Number **(B)** and diversity **(C)** of colonies counted after ~3 days. Error bars represent mean ± SEM. nd, not detected; *p < 0.05; **p < 0.01; ***p < 0.001, 1-tailed 1-sample *t*-tests vs. mean = 0 (vs. P/S, gray) or 2-tailed *t*-tests (FSW vs. TW, black). Figures represent 13 independent experiments.

All statistical tests were performed using GraphPad Prism (Figures 1, 6), JMP (Figure 3), or SPSS software (Figure 4).

## Results

### Saltwater Media Treatments Effectively Modify Bacterial Load

Low (i.e., 0.2 μm-filtered artificial seawater with penicillin and streptomycin; P/S) and high (i.e., artificial seawater with 20% adult-exposed tank water; AEW) bacterial load media were prepared from FSW as described. These treatments were not intended to be completely sterile or to replicate the natural environmental microbiota entirely, but rather to manipulate numbers of relevant exogenous bacteria and, in the case of AEW, to supply potentially relevant *S. purpuratus*-associated microbiota. We assayed numbers and diversity of culturable bacteria in these media alongside standard laboratory filtered artificial seawater (FSW) by plating and incubation on marine broth agar. pH did not vary appreciably between matched medium treatments (8.19 in P/S, 8.28 in FSW, ~8.26 in AEW; Supplementary Figure 1). Both number (CFU/μL) and diversity (distinct colony types) were significantly different between treatment groups (*p* < 0.0001, Kruskall–Wallis test) while variances were not (Bartlett's test). In *post-hoc* tests, the P/S treatment reduced culturable bacterial load over 3 days to undetectable, which was significantly different from the FSW (*p* < 0.05) and AEW treatments (*p* < 0.01; 1-tailed 1-sample *t*-tests, Figure 1). Conversely, the AEW treatment significantly increased both the number (mean 3.96 CFU/ μL, *p* < 0.01) and diversity (mean 4.42 distinct colony types/plate, *p* < 0.0001) of cultured colonies compared to normal FSW (mean 1.48 CFU/μL, 1.67 distinct colony types/plate, 2-tailed *t*-tests, Figure 1).

### Majority of Bacterial Load Is Acquired at the Feeding Larval Stage and Localized to the Gut Lumen

**Figure 2:**
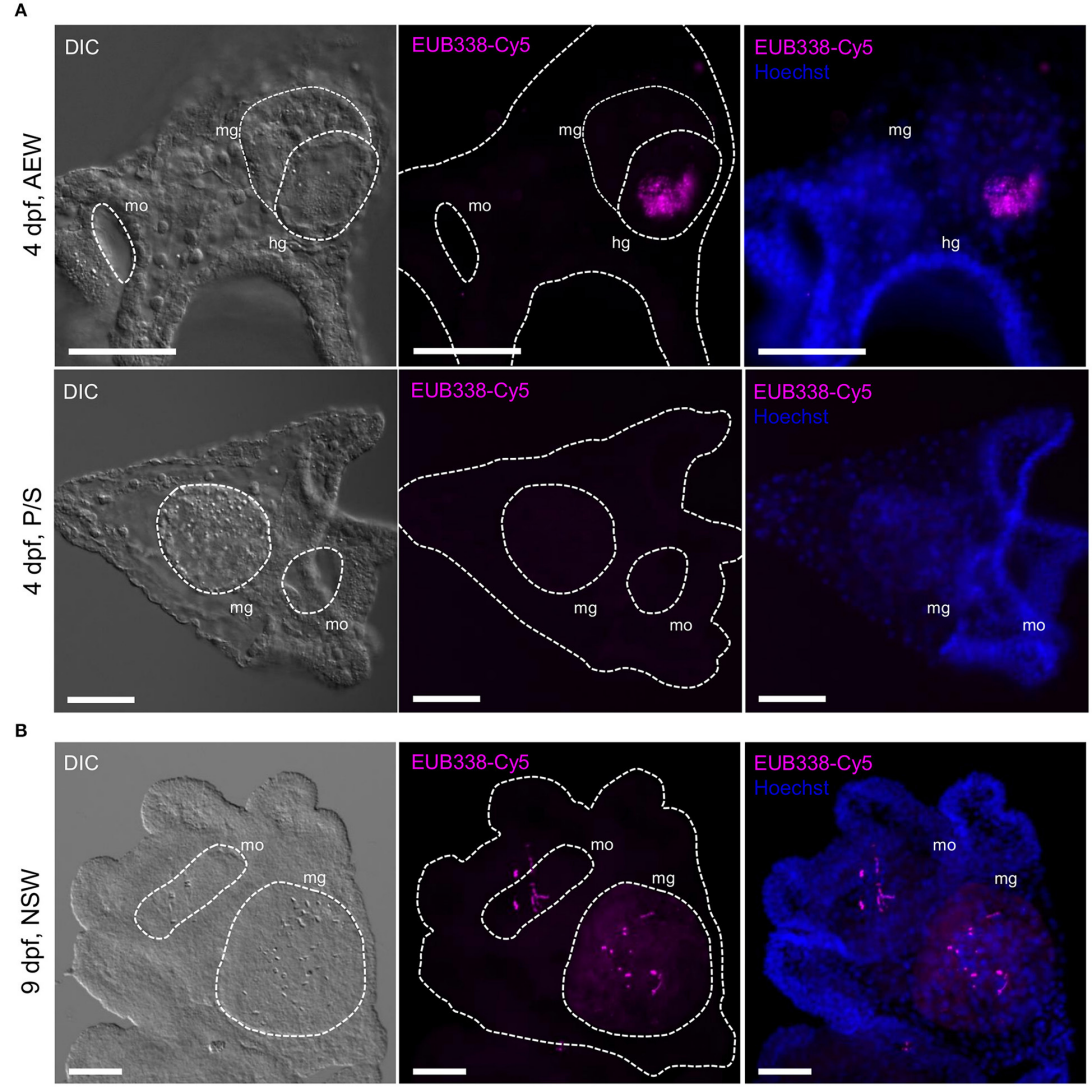
Bacterial microbiota are localized to the larval mouth and gut lumen. Bacterial colonization was visualized in 4-arm larvae using fluorescent 16S *in situ* hybridization. **(A)** 4 dpf laboratory-raised larvae grown in FSW + 20% 40 μm adult-exposed tank water (top, AEW) or 0.2 μm-filtered FSW + P/S (bottom, P/S). **(B)** 9 dpf larva raised in natural seawater. All scale bars = 50 μm. hg, hindgut; mg, midgut; mo, mouth. In the literature, the midgut and hindgut are also referred to as the stomach and intestine. Images are representative of >5 independent experiments.

We visualized bacterial colonization over the course of *S. purpuratus* embryonic and early larval development with *in situ* fluorescent hybridization using the pan-bacterial probe EUB338 conjugated to Cy5 (16S FISH) (53). The effectiveness and specificity of the probe were confirmed on individual and aggregated bacterial cells in both pure bacterial cultures and bacteria-associated larvae relative to a Cy5-conjugated scramble control (22). Preliminary 16S rRNA PCR experiments detected low levels of bacteria associated with eggs, but not sperm (not shown). In repeated staining experiments with EUB338-Cy5, eggs were sporadically associated with Cy5^+^ bacteria (19.4% of eggs Cy5^+^, *n* = 36, mean ± SD = 0.33 ± 0.93 Cy5^+^ spots/egg), even when collected and fixed directly from the gonopores (20.0% Cy5^+^, *n* = 15, 0.20 Cy5^+^ spots/egg) (Supplementary Figures 2A–D,F). Similarly, in a brood-matched time course experiment, 24 h post-fertilization (hpf) blastulae (28.1% Cy5^+^, *n* = 32, 1.22 ± 2.51 Cy5^+^ spots/blastula), and 48 hpf gastrulae appeared minimally colonized, regardless of the bacterial load in their environment (AEW, FSW, or P/S, Supplementary Figures 2E,F). Reduced, but not significantly different staining patterns were observed with the scramble control probe (12.5% Cy5^+^, *n* = 24, 0.29 ± 0.91 Cy5^+^ spots/egg). Conversely, by the time larvae are able to feed (4 dpf), Cy5^+^ bacteria are clearly visualized around the mouth, in the midgut and hindgut lumens (also referred to as the stomach and intestine, respectively), and, to a lesser degree, associated with the ectoderm (100% Cy5+, *n* = 11, 22.36 ± 13.86 Cy5^+^ spots/larva), particularly (but not significantly) when larvae are exposed to adult-associated bacteria (100% Cy5^+^, *n* = 6, 33.83 ± 11.11 Cy5^+^ spots/ larva) (Figure 2; Supplementary Figure 2F). Significantly fewer Cy5^+^ bacteria are seen associated with larvae raised in P/S, suggesting that, as expected, this treatment effectively reduces the larval microbiota (85.7% Cy5^+^, *n* = 7, 4.86 ± 5.40 Cy5^+^ spots/larva).

### The Laboratory Adult-Exposed and Natural Seawater Larval Microbiomes

We sampled zygotes, 4 dpf unfed larvae, and 11 dpf feeding larvae spawned and raised in adult-exposed tank water (AEW) or in natural seawater (NSW); their relevant culture media; and their associated *R. lens* feeding cultures. From these, we sequenced the 16S rRNA regions V3/V4 (55) using Illumina MiSeq and analyzed bacterial community composition with the microbial ecology program QIIME (59).

**Figure 3:**
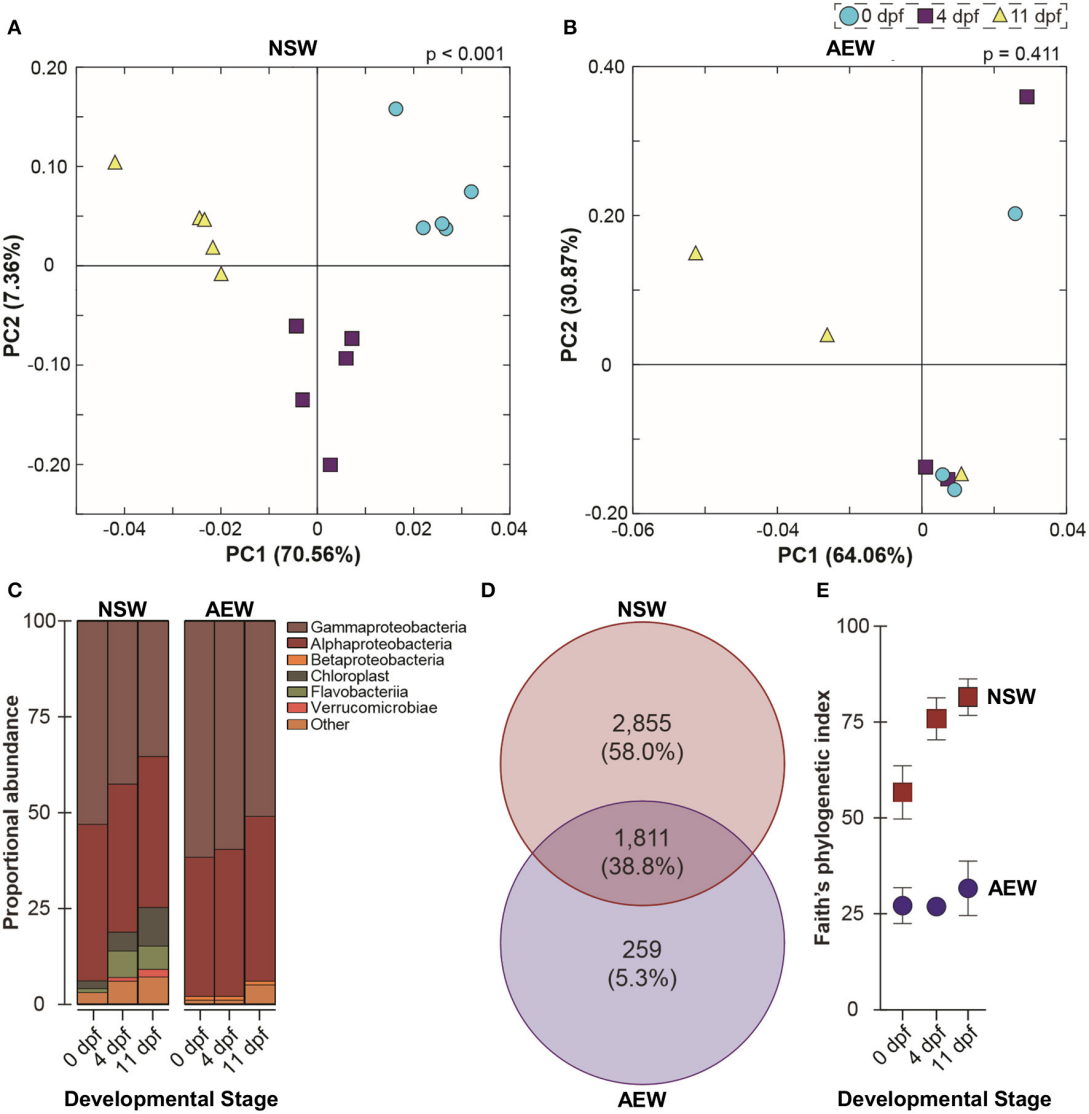
The natural seawater and laboratory microbiomes in early larval development. **(A,B)** UniFrac-weighted principal coordinate analysis of operational taxonomic units (OTUs) associated with zygotes, 4 dpf larvae, and 11 dpf larvae raised in natural seawater (NSW) and in the laboratory (AEW). **(C)** Relative class-level abundance profiles across early development in NSW- and AEW-exposed larvae. **(D)** Venn diagram illustrating NSW-specific, AEW-specific, and overlapping larva-associated bacterial OTUs. **(E)** Differential phylogenetic diversity (Faith's index) over early development in NSW- and AEW-reared zygotes and larvae.

At phylum level, the NSW- and AEW-raised larvae-associated bacterial communities were superficially similar (i.e., composed predominantly of Alpha and Gammaproteobacteria). Following fertilization, the bacterial communities associated with NSW-reared embryos and larvae shifted significantly in community membership (ANOSIM, unweighted UniFrac, *p* < 0.001) and structure (ANOSIM for weighted UniFrac, *p* < 0.001) while those of their laboratory-reared counterparts did not (ANOSIM, unweighted UniFrac, *p* = 0.129; ANOSIM, weighted UniFrac, *p* = 0.411) (Figures 3A,B,D; Supplementary Figure 3, Supplementary Table 1). This inconsistency appears linked to the fact that NSW- and AEW-exposed individuals recruited different (ANOSIM, *p* < 0.001 for all), yet host-specific (ANOSIM, *p* < 0.001 for all; Figures 3B,C; Supplementary Figure 4) bacterial communities.

Specifically, across developmental stages, 38.8% of bacterial OTUs were shared between NSW and AEW individuals, while 58.0% were NSW-specific and 5.3% were AEW-specific (Figure 3C). Moreover, the “natural” microbiota were taxonomically richer than the laboratory microbiota, with community diversity only increasing significantly with development in NSW (Figures 3A,B,D; Supplementary Figure 3, Supplementary Table 1). Differences in community patterning during development were also observed at higher taxonomic levels. For example, the relative abundance of Proteobacteria decreased (from 94.5 to 76.3%; one-way ANOVA, *p* > 0.0001) through to the larval stage for NSW animals while that for Bacteroidetes (from 1.6 to 7.4%; one-way ANOVA, *p* > 0.0001) and Cyanobacteria (from 1.9 to 9.6%; one-way ANOVA, *p* > 0.0001) increased (Figure 3B; Supplementary Table 1). A similar trend, namely, a temporal decrease in the relative abundance of Proteobacteria and an increase in the prevalence of “other” taxa, was observed in laboratory-raised larvae but it was not statistically significant (one-way ANOVA, *p* = 0.3134). A list of identified OTUs associated with zygotes and larvae in both environments is provided in Supplementary Table 1.

### Reduced Bacterial Load Is Associated With Accelerated Larval Growth

**Figure 4:**
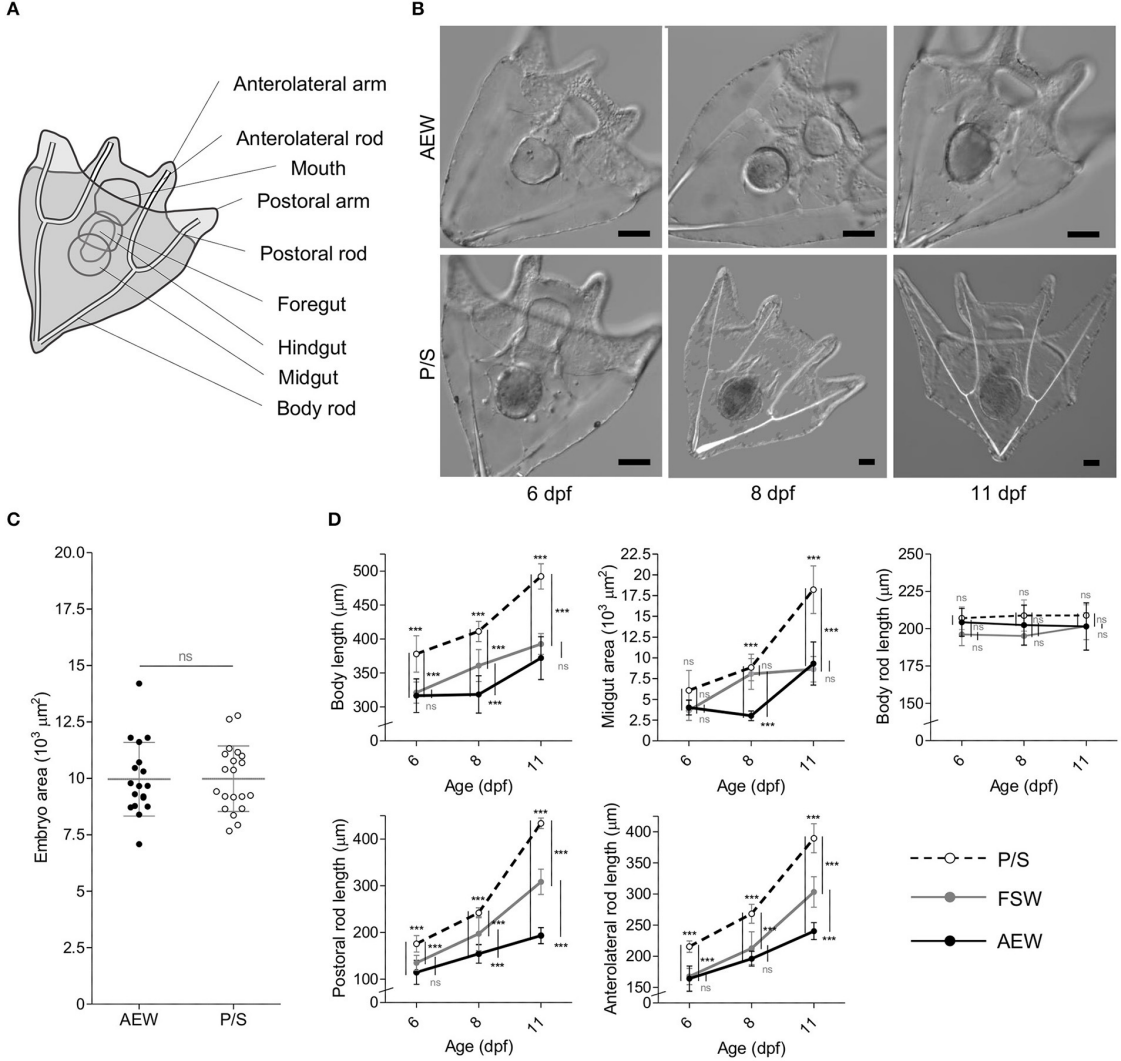
Bacterial load perturbation induces morphological plasticity in *S. purpuratus* larvae. **(A)** Schematic diagram of a 4-arm pluteus larva indicating the morphological measurements made in this study. **(B)** DIC images of 6–11 dpf AEW- and P/S-raised larvae. Scale bars = 50 μm. **(C)** Embryo size at 24 hpf. **(D)** Morphological measurements of P/S- FSW-, and AEW- raised larvae from 6–11 dpf. Statistics represent the results of a 2-tailed *t* test **(C)** or 2-way ANOVA with post-test Bonferroni testing between treatment pairs at each time point **(D)**. Error bars represent mean ± SD. ns, not significant (*p* > 0.05); ***p < 0.001.

Eggs and sperm were collected from the same mate pair directly into P/S, AEW, and normal FSW and washed, fertilized, and cultured in their respective media. At 24 hpf, no significant differences in embryo size (measured as area in μm^2^ from cross sections at maximum focus) were observed between groups (*p* = 0.9703, *n* = 38, 2-tailed *t*-test, Figure 4C). At 48 hpf, all animals observed were at mid-gastrula stage, and no morphological differences were observed between groups (Supplementary Figure 7). Similarly, at 4 dpf, all animals in each group had progressed to 4-arm larva stage, and no significant morphological differences were observed (not shown). Animals were fed at 5 dpf, and from 6–11 dpf, AEW-raised larvae consistently appeared smaller than P/S-raised larvae in every parameter measured [body length, midgut (or stomach) area, and the lengths of the post-oral and anterolateral rods] except body rod length, which typically does not grow during this time and is generally not phenotypically plastic (66) (Figures 4B,D). In each case, conventionally raised FSW larvae displayed a phenotype intermediate between AEW and P/S, consistent with this effect being a function of bacterial load and not an off-target effect of the antibiotics used. The same morphological differences have now been observed in three independent larval broods (Supplementary Figure 8). Furthermore, at 11 dpf, several animals in the P/S group could be observed budding spicules to advance to the 6-arm stage, consistent with an accelerated developmental trajectory (not shown). We also quantified these data by principal component analysis and ANOVA (Supplementary Table 2). Two components were extracted that together explain 92.971% of the total variance. Component 1 corresponded to body length, anterolateral rod length, postoral rod length, and midgut area; component 2 corresponded to body rod length.

### *Vibrio lentus* Induces Cellular Immune Responses, Disease, and Mortality in Sea Urchin Larvae

We reported previously that multiple *Splendidus* clade *Vibrio* species could be isolated from larvae, and, upon exposure to naive larvae, induce inflammatory or lethal responses (19, 22). In further experiments, similar colonies were isolated repeatedly from apparently healthy animals (though a subset showed signs of cellular immune activity) raised in both natural seawater (at MDI Biological Laboratory, Salsbury Cove, Maine, USA) (22) and in artificial seawater exposed to adult animals shipped from the Pacific coast in the laboratory (i.e., in AEW, this study). PCR amplification and sequencing of the 16S rRNA genes from these strains (V1 and V7, isolated from SRI and MDIBL, respectively) resulted in exact matches to multiple *Splendidus* clade *Vibrio*s and uncharacterized isolates using both BLASTn against all prokaryotic sequences and RDP (Supplementary Table 4). Therefore, we further characterized these isolates using *Vibrio*-specific primers targeting two widely-used barcoding genes, RNA polymerase sigma factor *rpoD* and transmembrane toxicity regulatory protein *toxR* (63) (Supplementary Table 3). Both *rpoD* nucleotide sequences gave best matches (≥96%) to *V. lentus* strain 3OM12. *toxR* resulted in a single match for each isolate, both to *V. lentus* strain CIP 107166 (67) (Supplementary Table 4). Therefore, we conclude that both V1 and V7 are strains of *V. lentus*.

**Figure 5:**
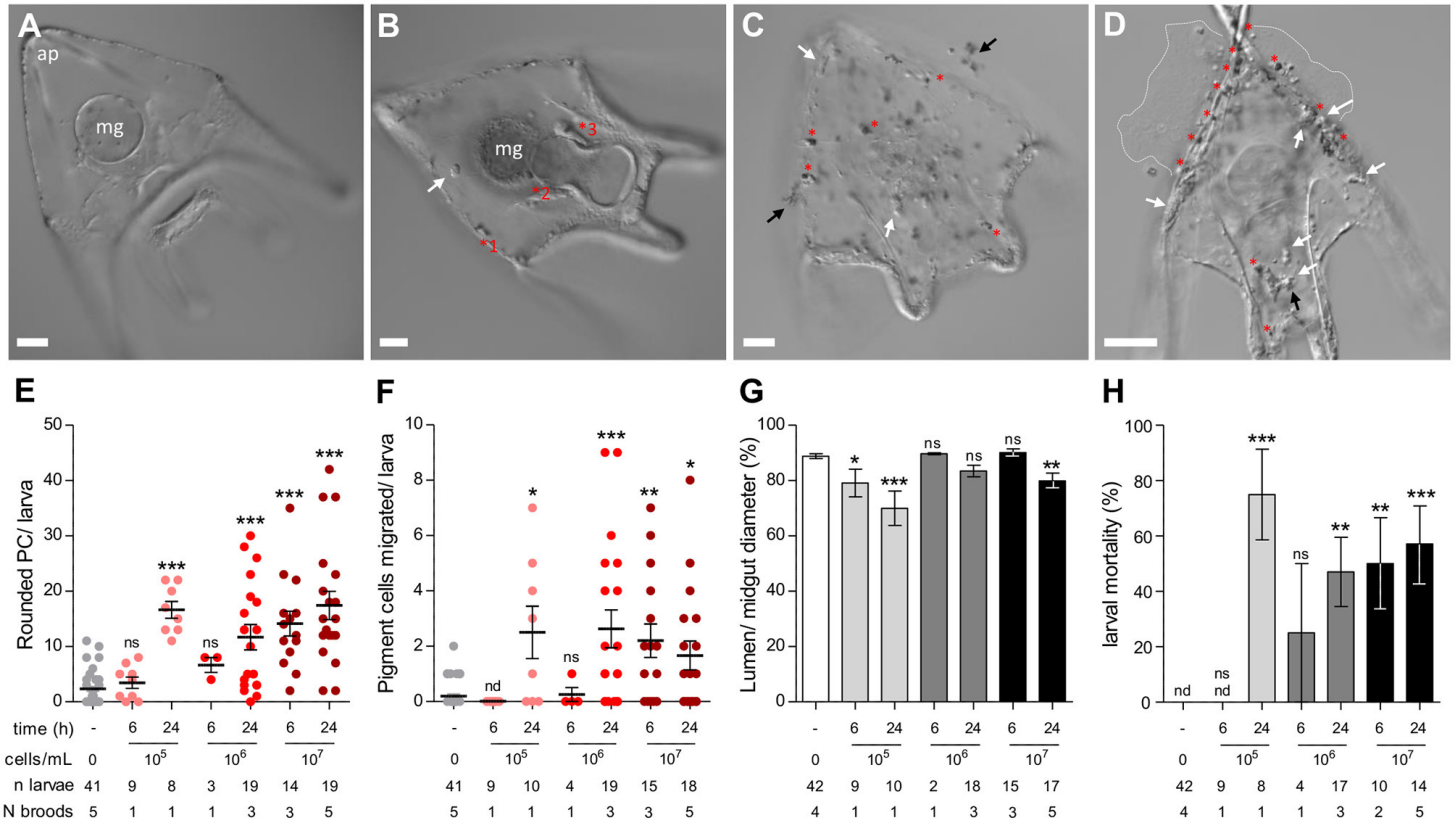
Cellular immune response and larval pathology during lethal *V. lentus* infection. **(A)** Unexposed larva. **(B)** Surviving larva at 2–3 days post exposure with residual immune activity. The midgut epithelium is hypertrophied and activated pigment cells are present at the ectoderm, pharyngeal/midgut epithelium (a.k.a. the esophageal and gastric epithelia), and coelomic pouch. A globular cell is patrolling the blastocoel. **(C)** Clumping and adherence of *V. lentus* to the larval ectoderm. Several globular cells and activated pigment cells are present at the ectoderm. **(D)** Skeletoectodermal penetration and larval lysis. The apical tips of the body rods have punctured the ectoderm. Many globular cells and activated pigment cells are present. Dashed white lines indicate the margins of lysed larval contents. **(E–G)** Quantification of changes in immune cell behavior in response to 10^5^, 10^6^, or 10^7^ *V. lentus* cells/mL at 6 and 24 h post exposure. **(H)** Larval mortality in response to 10^5^, 10^6^, or 10^7^ *V. lentus* cells/mL at 6 and 24 h post exposure. Lysed or skeletonized larvae were scored as dead. Scale bars = 50 μm. ap, apex; mg, midgut; red asterisks, activated pigment cells; black arrows, aggregated *V. lentus*; white arrows, globular cells. Error bars represent mean ± SEM. Statistics represent the results of Kruskal–Wallis with *post-hoc* Dunn's tests between experimental groups and unexposed controls. nd, not detected; ns; not significant, *p < 0.05; **p < 0.01, ***p < 0.001.

In preliminary experiments, we observed that both *V. lentus* strains were toxic to larvae. The dosage at which exposure becomes toxic or lethal seems to vary between broods, but based on repeated experiments, is between 10^5^ and 10^7^ cells/mL by 24 h (for V1, Figure 5H). Infection seems to progress through several stages. At dosages where the exposure is tolerated, a typical larval inflammatory response is observed, i.e., the midgut epithelium increases in size relative to its lumen and pigment cells activate, round, and migrate to the gut surface (Figures 5A,B,E,F) (22). In larvae that do not recover, the first signs of pathology are protrusion of the skeleton through the ectoderm at the arm tips and immune activation manifested as pigment cell activation/rounding (Figures 5C–H). Clumps of agglomerated *V. lentus* then become visible adhering to the larval ectoderm (Figure 5C), followed by ectodermal lysis (Figure 5D) and, eventually, skeletonization (Supplementary Figure 9).

### Developmental Exposure to Complex Microbiota Increases Larval Resistance to Lethal *Vibrio lentus* Infection

**Figure 6:**
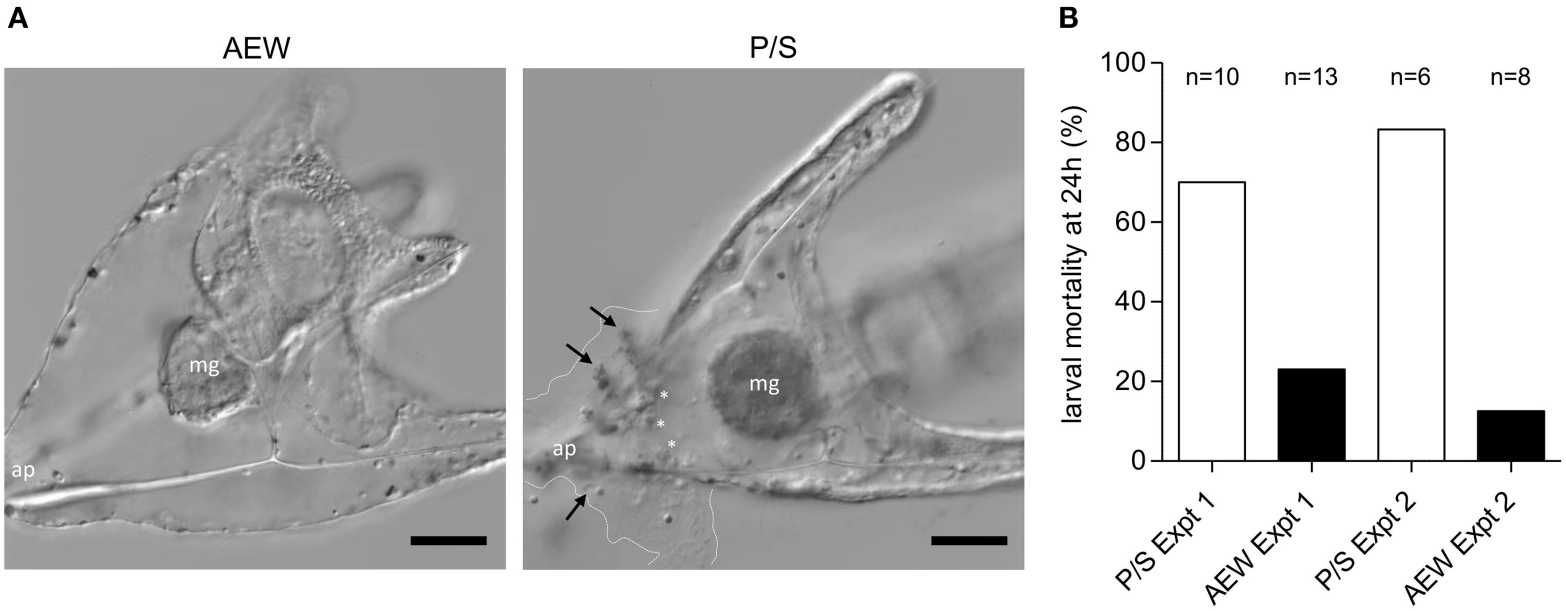
Bacterial exposure increases larval resistance to lethal *V. lentus* infection. AEW and P/S larvae were raised to 10 dpf and exposed to 10^7^ *V. lentus*/ mL for 24 h. **(A)** DIC images of 10 dpf AEW- (left) and P/S- (right) raised larvae at 24 h post-exposure. Dashed lines indicate margins of lysed ectodermal contents and bacterial invasion. Arrows indicate clumps of adherent *V. lentus*. White asterisks indicate globular cells. ap, apex; mg, midgut. **(B)** Larval mortality at 24 h post-exposure to *V. lentus*. Mortality was scored based on lysed ectoderm and/or exposed skeleton. Scale bars = 50 μm. Figures represent ~20 larvae/group observed in two independent experiments.

Larvae raised in high-microbial load natural seawater exhibit a high level of immune cell activity relative to typically lower microbial load conditions in the laboratory (22). This led us to question whether the developmental history of microbial exposure affects immune response efficiency. To assay immune function in AEW- and P/S-raised purple sea urchin larvae, we exposed larvae raised in these environments to 10^7^ *Vibrio lentus* cells/mL and observed them over 24 h. P/S-raised animals were transferred into FSW prior to infection. At 24 h post-exposure, AEW-raised larvae were more resistant to *V. lentus* exposure (Figures 6A,B). Most (~81% survival, *n* = 21) AEW-raised larvae appeared normal, while most (~25% survival, *n* = 16) P/S-raised larvae showed evidence of infection and/or tissue damage, including clouds of bacteria on the larval ectoderm, ectodermal lysis, blebbing cells and blastocoelar fluid, and disrupted or exposed skeletons (Figures 6A,B).

## Discussion

How and when marine invertebrate larvae are colonized by bacteria and other microbes, how this colonization interacts with developing immune systems, and how the bacterial microbiomes of laboratory- and NSW-raised larvae differ, are important considerations in our studies of larval immunity. Filling these gaps is important as studies in many other animal taxa suggest microbes play crucial, if not vital, roles in development (1–4). A growing body of evidence indicates this is also the case in echinoderms (31–33), and that cellular indications of immune activity in larva are increased in more complex natural seawater conditions relative to those typical of the laboratory (22). Previously, we isolated multiple associated bacterial strains from larvae raised in the laboratory and in artificial seawater in order to interrogate their interactions with the larval immune system (22, 24). In this study, we described the bacterial colonization dynamics of early embryos and larvae, compared the bacterial communities of NSW- and laboratory-raised larvae, and assayed the results of bacterial colonization on immune function against a larva-associated pathogen. Sterilizing FSW with 0.2 μm filtration and antibiotics and inoculating with adult-associated bacteria effectively modified environmental and larval bacterial load. While both eggs and early larvae are associated with significant bacterial diversity as determined by 16S sequencing, the vast majority of larval bacterial numbers are acquired at feeding and localized to the gut lumen. Importantly, this colonization had functional consequences on both morphological development and resistance to relevant environmental pathogens. These findings underscore reports in many other animal species (e.g., mammals) that bacterial exposure in early development is associated with immune development and function (14, 68).

### Kinetics of Bacterial Colonization

Associated bacteria have been characterized in diverse echinoderm taxa at many stages of development (22, 26–31), but spatiotemporal colonization kinetics in sea urchin larva remain poorly described (19, 69). Two likely sites for bacterial colonization are the larval digestive tract and the ectoderm, whether its external surface or the subcuticular space (70, 71). Walker and Lesse (72) used transmission electron microscopy to demonstrate the presence of rod-shaped, Gram-negative bacteria in the subcuticular spaces of larvae, juveniles, and associated adult tissue in the brooding, planktotrophic brittlestar *Amphimpholis squamata*. Cerra et al. (73) similarly visualized such rods in the hyaline layers of 7 dpf *Patiriella calcar* sea star larvae. Notably, in that study, bacteria were occasionally seen on the outer ectodermal surface, but subcuticular colonization was sporadic, non-synchronous, and not observed before larval stage. These bacteria were also observed in epithelial phagocytic vesicles, implying host interaction with this population.

In this study, 16S FISH revealed detectable, but very low numbers of bacteria associated with *S. purpuratus* eggs and embryos. In all cases in the embryo, bacteria appeared to be external to the hyaline layer and not internalized (Supplementary Figure 2). In contrast, depending on the bacterial load of the culture water, ~9–36-fold increases in stained bacteria were observed at the onset of the feeding larval stage vs. 24 hpf, almost entirely localized to the gut lumen (Figure 2; Supplementary Figure 2). These results suggest that while bacteria may associate with developing *S. purpuratus* externally, the primary site of colonization is the larval gut lumen.

Previous work reported that eggs are sterile when extruded from the gonopores (74). However, sterility was tested using culture in marine broth (75) and DNA staining, and it is unclear whether unculturable and unstained microbes may have been present (76). *S. purpuratus eggs* are coated in jelly, which likely prevents bacteria from reaching the egg surface (77). Majeske et al. (78) demonstrated expression of the immune response protein 185/333 (now SpTransformer) in oocytes within the adult *S. purpuratus ovary* and speculated that it may be secreted with cortical granules at fertilization. This and other immune programs could limit bacterial viability at the egg surface, and the rapidly emerging fertilization envelope would then provide barrier function until hatching. Consequently, three antimicrobial peptides, strongylocins 1, 2, and centrocin, begin to be expressed at early and 6-arm larval stages in the green sea urchin *S*. *droebachiensis*, respectively, perhaps coinciding with new immune challenges associated with feeding (79).

### Larval 16S Sequence Diversity in the Laboratory and Natural Seawater

In the laboratory, extreme precautions are usually taken to reduce microbial contamination; by design, field experiments are the opposite. Here, we attempted to raise and colonize larvae in artificial seawater with a microbiota derived from their wild-caught parents and compare typical microbiomes of early larvae in both environments. While larvae in the wild are colonized in an environment connected to, but different than, their benthic adult habitat, the adult microbiota should nonetheless include species that are capable of forming echinoderm associations. The laboratory and natural microbiomes have several qualitative similarities: both are predominantly Alphaproteobacteria and Gammaproteobacteria, with minimal diversity at zygote stage and increasing diversity over time (significant in NSW, not in AEW; Figure 3; Supplementary Figures 3, 5, 6, Supplementary Table 1). Additionally, larvae in both environments were hosts to bacterial communities significantly different from the surrounding seawater and from their *Rhodomonas* feeding cultures, suggesting a degree of selective colonization (Supplementary Figure 4) (28, 29, 31). This effect may be related to the increased alkalinity of the larval gut relative to their environment (80), as bacteria able to thrive in such environments could be positively selected. The current analysis did not address this possibility, but it should be explored in future studies. Regardless, community diversity was significantly reduced in AEW vs. the field (Figures 3B,D; Supplementary Table 1), a result observed across the animal kingdom (32, 33). Importantly, while seemingly incomplete, this experimental colonization was associated with downstream morphological and immune consequences (Figures 4, 6).

In this study, bacterial sequence diversity was detected in both laboratory- and ocean water-reared zygotes, but EUB338-Cy5^+^ bacteria were not observed in substantial numbers until larvae were several days old, and were localized almost entirely to the gut lumen (Figures 2, 3; Supplementary Figure 2). A similar result was recently observed in larvae of the polychaete worm *Hydroides elegans* (81). It is likely that loosely associated surface microbes are lost during washing and fixation—larvae were intentionally washed to remove nonadherent bacteria prior to imaging, as Cy5^+^ surface-associated bacteria are retained in similarly washed and fixed larvae exposed under laboratory conditions [e.g., to *V. diazotrophicus*; (22)]. However, staining visualizes bacteria associated with single animals, while sequencing samples are pooled from many animals. Therefore, bacterial colonization in *S. purpuratus* embryos most likely occurs from fertilization onward, but sporadically among individuals, and at a dramatically reduced rate until the onset of feeding, when the gut proper can be colonized *en masse*. It should be noted that older larvae may exhibit more complex ectodermal association as this study focused generally on larvae that were <14 days postfertilization. Future studies should investigate the potential presence of less intimate bacterial associations with the egg surface.

### Bacterial Exposure-Associated Morphological Plasticity

Morphological plasticity is well-described in echinoderm larvae (36, 37, 39) [Reviewed in (34, 35)]. In this study, we found that the sizes of multiple larval structures were associated with differences in bacterial exposure during development. At first glance, this effect appeared similar to feeding-associated phenotypic plasticity, as planktotrophic larvae consume bacteria, and gut bacteria are associated with digestive and metabolic functions in countless taxa (2, 3, 7, 8), including larval (38, 82) and adult sea urchins (27, 69). However, feeding-associated plasticity typically manifests as reduced gut size and increased arm length relative to body length under low food conditions, and our low bacterial load (P/S) animals were larger in all measurements (body length, gut area, post-oral arm length, anterolateral arm length) besides the body rod, which is not typically plastic in response to food (83). One explanation is that bacterial exposure mediates a trade-off between growth and immune competence during development, as predicted in Carrier et al. (82). In this model, larvae expend energy to establish and maintain a molecular and/or cellular immune state that would otherwise be diverted to growth and development in the absence of bacteria. As shown, the onset of feeding is associated with a stark increase in associated bacteria, primarily localized to the gut lumen.

Another potential explanation is the antibiotics used to reduce bacterial load causing β-lactam and/or aminoglycoside-associated off-target effects, e.g., mitochondrial inhibition (84) on larvae and or their food. Penicillin and streptomycin are commonly used, in sea urchin and other echinoderm embryo studies, to control bacterial contamination during rowing and microinjection [e.g., (24, 85)]. While our data do not address these hypotheses directly, the observed growth trends go in the opposite direction (antibiotic-exposed larvae are larger rather than smaller), and FSW-raised larvae exhibited an intermediate phenotype coincident with intermediate bacterial load. Furthermore, differences in food quality were not readily observed when cultured in FSW vs. PS, and feeding was normalized by counting viable cells in each culture separately. However, future work should investigate further the cellular, metabolic, and immune mechanisms presumably underlying this phenomenon.

### Pathology and Immune Response to *Vibrio lentus* Infection

Many *Splendidus* clade *Vibrio* strains are toxic to a wide array of both vertebrate and invertebrate aquatic organisms (43–51). Previously, we showed that several *Splendidus* clade *Vibrio* species could cause inflammation or death in purple sea urchin larvae (19, 22). Here, we observed the progression of infection, disease, and mortality in larvae exposed to *V. lentus*, which we have isolated from animals raised both in the laboratory in AEW and natural seawater. In tolerant animals, immune cell behaviors typical during exposure to the established model pathogen *V. diaztrophicus* are evident, namely midgut (or stomach) epithelial hypertrophy and pigment cell rounding and migration. However, at concentrations ≥10^5^ cells/mL, bacteria begin to swarm the larval ectoderm, and by 6–24 h, substantial tissue damage, lysis, and, eventually, skeletonization occur (Figure 5; Supplementary Figure 9). In these animals, numerous pigment and globular cells are observed at the ectodermal-bacterial interface, likely performing antimicrobial effector or wound repair functions.

In the octopus *Octopus vulgaris, V. lentus* infection is associated with the formation of surface lesions, deterioration of the skin, colonization of the internal organs, and death (48), consistent with the presentation of larvae exposed to their associated strains. Interestingly, the octopus-associated *lentus* strains tested did not cause disease after injection into sea bream or turbot, perhaps suggesting a degree of host specificity. A subsequent study (2006) demonstrated that *Vibrio splendidus*- *Vibrio lentus* related strains were able to cause disease in both octopus and fish via secretion of an extracellular protease. Conversely, Rojas et al. (47) observed similar effects on *Agropecten purpuratus* scallop larvae at 24 h exposure to multiple *V. splendidus* strains (bacterial adherence and tissue digestion). However, cell-free extracellular products heated or digested with proteinase K still induced substantial mortality, indicating that *Splendidus* clade *Vibrios* can cause host damage through both protein and non-protein secretions. Future work will elucidate the mechanisms by which *V. lentus* infect and kill sea urchin larvae and how larvae become resistant.

### The Larval Microbiota and the Antibacterial Immune Response

We observed that bacterial exposure in early development resulted in increased resistance to lethal *V. lentus* infection. Although numbers encountered in the ocean are probably much lower than the doses used in this study, this effect is interesting as *Vibrio* spp., including *Splendidus* clade *Vibrios*, are regularly associated with healthy sea urchin larvae in the lab and in the field [(22), this study]. This may represent a form of opportunism (86) where *Vibrio* spp. are normally commensal or mutualistic but can overwhelm larval defenses under conditions of excessive numbers or an underdeveloped antibacterial immune response. Reduction of the normal bacterial flora in the gut lumen may present a niche into which *Vibrio* can expand and thereby causes disease. However, since the site of infection in this model is the ectoderm, rather than the gut, it seems more likely that the increased susceptibility of low bacterial load larvae to *V. lentus* infection represents impaired development or education of the immune system. Regardless, the ease with which dysbiosis can induce toxicity or death in the host suggests that proper colonization and immune co-development are crucial for establishing a functional host-microbiota relationship. Future studies should clarify the prevalence of *Splendidus* clade *Vibrio* association and characterize the immune and developmental mechanisms that confer resistance.

## Conclusions and Future Directions

In this report, we have characterized the spatiotemporal kinetics of bacterial colonization in early purple sea urchins in order to understand further how microbiota and antibacterial immune states are simultaneously established in development. Using 16S-FISH, bacteria are present, but scarce, on the egg and early embryo surface, and a large flux of bacteria become associated with larvae at the onset of feeding. Bacterial exposure in early development is associated with reduced larval growth but seems crucial to establishing competent immune responses against *V. lentus* and likely other commonly encountered opportunistic pathogens. Future work should identify the pathways and mechanisms through which bacteria participate in growth, development, and immunity.

## Data Availability Statement

The raw 16S sequence reads as part of this dataset are available on Dryad at https://doi.org/10.5061/dryad.qv9s4mw9v. Laboratory (V1) and MDIBL (V7) *V. lentus* strain 16S (accession numbers MN365723, MN365724), *rpoD* (MN371278, MN371279), and *toxR* (MN371280, MN371281) sequences have been uploaded to GenBank. The remaining raw datasets supporting the conclusions of this manuscript are either included in the figures or Supplementary Data or will be made available to any qualified researchers upon request.

## Ethics Statement

Animal protocols were approved by the Sunnybrook Research Institute Animal Care Committee.

## Author Contributions

NS designed the research, collected samples, performed experiments and data analysis, and wrote the manuscript. TC collected samples, performed experiments and data analysis, and edited the manuscript. CS collected samples and performed experiments. AR provided funding and edited the manuscript. AH provided funding, performed data analysis, and wrote the manuscript. JR designed the research, provided funding, collected samples, performed experiments and data analysis, and wrote the manuscript.

### Conflict of Interest

The authors declare that the research was conducted in the absence of any commercial or financial relationships that could be construed as a potential conflict of interest.

## Abbreviations

FSW: artificial filtered seawater
P/S: 0.2 μm-filtered artificial seawater + 100 U/mL penicillin, 100 μg/mL streptomycin
AEW: artificial filtered seawater + 20% (v/v) adult-exposed tank water
CFU: colony-forming unit
dpf: days post fertilization
NSW: natural seawater
hpf: hours post fertilization
OTU: operational taxonomic unit
PCoA: principle coordinate analysis.

## References

[1] McFall-Ngai MJ. Unseen forces: the influence of bacteria on animal development. Dev Biol. (2002) 242:1–14. 10.1006/dbio.2001.0522 11795936

[2] Gilbert SF Sapp J Tauber AI. A symbiotic view of life: we have never been individuals. Q Rev Biol. (2012) 87:325–41. 10.1086/668166 23397797

[3] Mcfall-Ngai M Hadfield MG Bosch TC Carey HV Domazet-Loso T Douglas AE. Animals in a bacterial world, a new imperative for the life sciences. Proc Natl Acad Sci USA. (2013) 110:3229–36. 10.1073/pnas.1218525110 23391737 PMC3587249

[4] Parker A Lawson MAE Vaux L Pin C. Host-microbe interaction in the gastrointestinal tract. Environ Microbiol. (2018) 20:2337–53. 10.1111/1462-2920.13926 28892253 PMC6175405

[5] Ruby EG. Lessons from a cooperative, bacterial-animal association: the *Vibrio fischeri*–*Euprymna scolopes* light organ symbiosis. Ann Rev Microbiol. (1996) 50:591–624. 10.1146/annurev.micro.50.1.591 8905092

[6] Kremer N Koch EJ El Filali A Zhou L Heath-Heckman EAC Ruby EG. (2018). persistent interactions with bacterial symbionts direct mature-host cell morphology and gene expression in the Squid-Vibrio Symbiosis. mSystems. 3, 1–17. 10.1128/mSystems.00165-18 30320217 PMC6172772

[7] Donia MS Fricke WF Partensky F Cox J Elshahawi SI White JR. Complex microbiome underlying secondary and primary metabolism in the tunicate-Prochloron symbiosis. Proc Natl Acad Sci USA. (2011) 108:E1423–32. 10.1073/pnas.1111712108 22123943 PMC3251135

[8] Slaby BM Hackl T Horn H Bayer K Hentschel U. Metagenomic binning of a marine sponge microbiome reveals unity in defense but metabolic specialization. ISME J. (2017) 11:2465–78. 10.1038/ismej.2017.101 28696422 PMC5649159

[9] Rosenberg E Koren O Reshef L Efrony R Zilber-Rosenberg I. The role of microorganisms in coral health, disease and evolution. Nat Rev Microbiol. (2007) 5:355–62. 10.1038/nrmicro1635 17384666

[10] Webster NS. Sponge disease: a global threat? Environ Microbiol. (2007) 9:1363–75. 10.1111/j.1462-2920.2007.01303.x 17504474

[11] Rubanov A Russell KA Rothman JA Nieh JC Mcfrederick QS. Intensity of *Nosema ceranae* infection is associated with specific honey bee gut bacteria and weakly associated with gut microbiome structure. Sci Rep. (2019) 9:3820. 10.1038/s41598-019-40347-6 30846803 PMC6405881

[12] Carding S Verbeke K Vipond DT Corfe BM Owen LJ. Dysbiosis of the gut microbiota in disease. Microb Ecol Health Dis. (2015) 26:26191. 10.3402/mehd.v26.26191 25651997 PMC4315779

[13] Sánchez B Gueimonde M Pena AS Bernardo D. Intestinal microbiota as modulators of the immune system. J Immunol Res. (2015) 2015:159094. 10.1155/2015/159094 25821835 PMC4363913

[14] Fiebiger U Bereswill S Heimesaat MM. Dissecting the interplay between intestinal microbiota and host immunity in health and disease: lessons learned from germfree and gnotobiotic animal models. Eur J Microbiol Immunol. (2016) 6:253–71. 10.1556/1886.2016.00036 27980855 PMC5146645

[15] Eberl G. The microbiota, a necessary element of immunity. C R Biol. (2018) 341:281–3. 10.1016/j.crvi.2018.03.003 29631890

[16] Mcclay DR. Evolutionary crossroads in developmental biology: sea urchins. Development. (2011) 138:2639–48. 10.1242/dev.048967 21652646 PMC3109595

[17] Buckley KM Rast JP. An organismal model for gene regulatory networks in the gut-associated immune response. Front Immunol. (2017) 8:1297. 10.3389/fimmu.2017.01297 29109720 PMC5660111

[18] Ettensohn CA “Sea urchins as a model system for studying embryonic development,” In: Caplan MJ, editor. Reference Module in Biomedical Sciences. Amsterdam: Elsevier (2017).

[19] Heyland A Schuh N Rast J. Sea urchin larvae as a model for postembryonic development. Results Probl Cell Differ. (2018) 65:137–61. 10.1007/978-3-319-92486-1_8 30083919

[20] Metchnikoff I Lectures on the Comparative Pathology of Inflammation: Delivered at the Pasteur Institute in 1891. London: Kegan Paul, Trench, Trübner & Co., Ltd (1893).

[21] Solek CM Oliveri P Loza-Coll M Schrankel CS Ho EC Wang G. An ancient role for Gata-1/2/3 and Scl transcription factor homologs in the development of immunocytes. Dev Biol. (2013) 382:280–92. 10.1016/j.ydbio.2013.06.019 23792116

[22] Ho EC Buckley KM Schrankel CS Schuh NW Hibino T Solek CM Perturbation of gut bacteria induces a coordinated cellular immune response in the purple sea urchin larva. Immunol Cell Biol. (2016) 94:861–74. 10.1038/icb.2016.51 27192936 PMC5073156

[23] Schrankel CS Solek CM Buckley KM Anderson MK Rast JP. A conserved alternative form of the purple sea urchin HEB/E2–2/E2A transcription factor mediates a switch in E-protein regulatory state in differentiating immune cells. Dev Biol. (2016) 416:149–61. 10.1016/j.ydbio.2016.05.034 27265865

[24] Buckley KM Ho ECH Hibino T Schrankel CS Schuh NW Wang G. IL17 factors are early regulators in the gut epithelium during inflammatory response to Vibrio in the sea urchin larva. Elife. (2017) 6:e23481. 10.7554/eLife.23481.025 28447937 PMC5457136

[25] Cammarata M Palgliara P Elie Metchnikoff and the multidisciplinary link novelty among zoology, embryology and innate immunity. Invertebr Surv J. (2018) 15:234–9. 10.25431/1824-307X/isj.v15i1.234-239

[26] Galac MR Bosch I Janies DA Bacterial communities of oceanic sea star (Asteroidea: Echinodermata) larvae. Mar Biol. (2016) 163:162 10.1007/s00227-016-2938-3

[27] Hakim JA Koo H Kumar R Lefkowitz EJ Morrow CD Powell ML. The gut microbiome of the sea urchin, *Lytechinus variegatus*, from its natural habitat demonstrates selective attributes of microbial taxa and predictive metabolic profiles. FEMS Microbiol Ecol. (2016) 92:fiw146. 10.1093/femsec/fiw146 27368709 PMC5975844

[28] Carrier TJ Reitzel AM. Convergent shifts in host-associated microbial communities across environmentally elicited phenotypes. Nat Commun. (2018) 9:952. 10.1038/s41467-018-03383-w 29507332 PMC5838112

[29] Carrier TJ Wolfe K Lopez K Gall M Janies DA Byrne M Diet-induced shifts in the crown-of-thorns (*Acanthaster* sp.) larval microbiome. Mar Biol. (2018) 165:157 10.1007/s00227-018-3416-x

[30] Jackson EW Pepe-Ranney C Debenport SJ Buckley DH Hewson I. The microbial landscape of sea stars and the anatomical and interspecies variability of their microbiome. Front Microbiol. (2018) 9:1829. 10.3389/fmicb.2018.01829 30150973 PMC6099117

[31] Carrier TJ Reitzel A Bacterial community dynamics during embryonic and larval development of three confamilial echinoids. Mar Ecol Prog Ser. (2019) 166:179–88. 10.3354/meps12872

[32] Kohl KD Carey HV. A place for host-microbe symbiosis in the comparative physiologist's toolbox. J Exp Biol. (2016) 219:3496–504. 10.1242/jeb.136325 27852759

[33] Carrier TJ Reitzel AM. The hologenome across environments and the implications of a host-associated microbial repertoire. Front Microbiol. (2017) 8:802. 10.3389/fmicb.2017.00802 28553264 PMC5425589

[34] Miner BG Mechanisms underlying feeding-structure plasticity in echinoderm larvae. In: Flatt T Heyland A, editors. Mechanisms of Life History Evolution. Oxford: Oxford University Press (2011). p. 221–9.

[35] Mcalister JS Miner BG Phenotypic plasticity of feeding structures in marine invertebrate larvae. In: Carrier TJ Reitzel AM Heyland A, editors. Evolutionary Ecology of Marine Invertebrate Larvae. Oxford: Oxford University Press (2018). p. 103–23.

[36] Strathmann RR Fenaux L Strathmann MF. Heterochronic developmental plasticity in larval sea urchins and its implications for evolution of nonfeeding larvae. Evolution. (1992) 46:972–86. 10.1111/j.1558-5646.1992.tb00613.x 28564401

[37] Reitzel AM Heyland A Reduction in morphological plasticity in echinoid larvae: relationship of plasticity with maternal investment and food availability. Evol Ecol Res. (2007) 9:109–21.

[38] Carrier TJ King BL Coffman JA. Gene expression changes associated with the developmental plasticity of sea urchin larvae in response to food availability. Biol Bull. (2015) 228:171–80. 10.1086/BBLv228n3p171 26124444 PMC4706744

[39] Kalachev AV Yurchenko OV Osten VG Phenotypic plasticity in pre-feeding larvae of sea urchins, *Mesocentrotus nudus* and *Strongylocentrotus intermedius*. Invertebr Zool. (2018) 15:420–33. 10.15298/invertzool.15.4.09

[40] Gilles KW Pearse JS Disease in sea urchin *Strongylocentrotus purpuratus*: experimental infection and bacterial virulence. Dis Aquat Org. (1986) 1:105–14. 10.3354/dao001105

[41] Bonnin-Jusserand M Copin S Le Bris C Brauge T Gay M Brisabois A. Vibrio species involved in seafood-borne outbreaks (*Vibrio cholerae, V. parahaemolyticus* and *V. vulnificus*): review of microbiological versus recent molecular detection methods in seafood products. Crit Rev Food Sci Nutr. (2019) 59:597–610. 10.1080/10408398.2017.1384715 28956623

[42] Guerinot ML West PA Lee JV Colwell RR *Vibrio diazotrophicus* sp. nov, a Marine nitrogen-fixing bacterium. Int J Syst Bacteriol. (1982) 32:350–7. 10.1099/00207713-32-3-350

[43] Gay M Renault T Pons AM Le Roux F. Two vibrio splendidus related strains collaborate to kill *Crassostrea gigas*: taxonomy and host alterations. Dis Aquat Organ. (2004) 62:65–74. 10.3354/dao062065 15648832

[44] Nicolas JL Corre S Gauthier G Robert R Ansquer D Bacterial problems associated with scallop *Pecten maximus* larval culture. Dis Aquat Org. (1996) 27:67–76. 10.3354/dao027067

[45] Gómez-León J Villamil L Lemos ML Novoa B Figueras A. Isolation of *Vibrio alginolyticus* and *Vibrio splendidus* from aquacultured carpet shell clam (*Ruditapes decussatus*) larvae associated with mass mortalities. Appl Environ Microbiol. (2005) 71:98–104. 10.1128/AEM.71.1.98-104.2005 15640176 PMC544237

[46] Domeneghetti S Varotto L Civettini M Rosani U Stauder M Pretto T. Mortality occurrence and pathogen detection in *Crassostrea gigas* and *Mytilus galloprovincialis* close-growing in shallow waters (Goro lagoon, Italy). Fish Shellfish Immunol. (2014) 41:37–44. 10.1016/j.fsi.2014.05.023 24909498

[47] Rojas R Miranda CD Opazo R Romero J. Characterization and pathogenicity of *Vibrio splendidus* strains associated with massive mortalities of commercial hatchery-reared larvae of scallop *Argopecten purpuratus* (Lamarck, 1819). J Invertebr Pathol. (2015) 124:61–9. 10.1016/j.jip.2014.10.009 25450196

[48] Farto R Armada SP Montes M Guisande JA Pérez MJ Nieto TP. *Vibrio lentus* associated with diseased wild octopus (*Octopus vulgaris*). J Invertebr Pathol. (2003) 83:149–56. 10.1016/S0022-2011(03)00067-3 12788284

[49] Gatesoupe FJ Lambert C Nicolas JL. Pathogenicity of *Vibrio splendidus* strains associated with turbot larvae, *Scophthalmus maximus*. J Appl Microbiol. (1999) 87:757–63. 10.1046/j.1365-2672.1999.00922.x 10594718

[50] Farto R Armada SP Montes M Perez MJ Nieto TP. Presence of a lethal protease in the extracellular products of *Vibrio splendidus*. J Fish Dis. (2006) 29:701–7. 10.1111/j.1365-2761.2006.00746.x 17169102

[51] Lasa A Avendano-Herrera R Estrada JM Romalde JL. Isolation and identification of *Vibrio toranzoniae* associated with diseased red conger eel (*Genypterus chilensis*) farmed in Chile. Vet Microbiol. (2015) 179:327–31. 10.1016/j.vetmic.2015.06.003 26072371

[52] Zobell CE Studies on marine bacteria. I. The cultural requirements of heterotrophic aerobes. J Mar Res. (1941) 4:42–75.

[53] Amann RI Binder BJ Olson RJ Chisholm SW Devereux R Stahl DA. Combination of 16S rRNA-targeted oligonucleotide probes with flow cytometry for analyzing mixed microbial populations. Appl Environ Microbiol. (1990) 56:1919–25. 2200342 10.1128/aem.56.6.1919-1925.1990 PMC184531

[54] Stahl DA Amann RI Development and application of nucleic acid probes. In: Stackebrandt E Goodfellow M, editors. Nucleic Acid Techniques in Bacterial Systematics. Chichester: John Wiley & Sons (1991). p. 205–48.

[55] Klindworth A Pruesse E Schweer T Peplies J Quast C Horn M. Evaluation of general 16S ribosomal RNA gene PCR primers for classical and next-generation sequencing-based diversity studies. Nucleic Acids Res. (2013) 41:e1. 10.1093/nar/gks808 22933715 PMC3592464

[56] Zhang J Kobert K Flouri T Stamatakis A. PEAR: a fast and accurate illumina paired-end reAd mergeR. Bioinformatics. (2014) 30:614–20. 10.1093/bioinformatics/btt593 24142950 PMC3933873

[57] Bolger AM Lohse M Usadel B. Trimmomatic: a flexible trimmer for illumina sequence data. Bioinformatics. (2014) 30:2114–20. 10.1093/bioinformatics/btu170 24695404 PMC4103590

[58] Edgar RC Haas BJ Clemente JC Quince C Knight R. UCHIME improves sensitivity and speed of chimera detection. Bioinformatics. (2011) 27:2194–200. 10.1093/bioinformatics/btr381 21700674 PMC3150044

[59] Caporaso JG Kuczynski J Stombaugh J Bittinger K Bushman FD Costello EK. QIIME allows analysis of high-throughput community sequencing data. Nat Methods. (2010) 7:335–6. 10.1038/nmeth.f.303 20383131 PMC3156573

[60] Lozupone C Knight R. UniFrac: a new phylogenetic method for comparing microbial communities. Appl Environ Microbiol. (2005) 71:8228–35. 10.1128/AEM.71.12.8228-8235.2005 16332807 PMC1317376

[61] Schindelin J Arganda-Carreras I Frise E Kaynig V Longair M Pietzsch T. Fiji: an open-source platform for biological-image analysis. Nat Methods. (2012) 9:676–82. 10.1038/nmeth.2019 22743772 PMC3855844

[62] Watanabe Y Shinzato N Fukatsu T. Isolation of actinomycetes from termites' guts. Biosci Biotechnol Biochem. (2003) 67:1797–801. 10.1271/bbb.67.1797 12951516

[63] Pascual J Macian MC Arahal DR Garay E Pujalte MJ. Multilocus sequence analysis of the central clade of the genus *Vibrio* by using the 16S rRNA, recA, pyrH, rpoD, gyrB, rctB and toxR genes. Int J Syst Evol Microbiol. (2010) 60:154–65. 10.1099/ijs.0.010702-0 19648344

[64] Hall TA BioEdit: a user-friendly biological sequence alignment editor and sequence analysis program for Windows 95/98/NT. Nucleic Acids Symp Ser. (1999) 41:95–8.

[65] Wang Q Garrity GM Tiedje JM Cole JR. Naïve Bayesian Classifier for rapid assignment of rRNA sequences into the new bacterial taxonomy. Appl Environ Microbiol. (2007) 73:5261–7. 10.1128/AEM.00062-07 17586664 PMC1950982

[66] Miner BG Evolution of feeding structure plasticity in marine invertebrate larvae: a possible trade-off between arm length and stomach size. J Exp Mar Biol Ecol. (2005) 315:117–25. 10.1016/j.jembe.2004.09.011

[67] Le Roux F Goubet A Thompson FL Faury N Gay M Swings J. *Vibrio gigantis* sp. nov, isolated from the haemolymph of cultured oysters (*Crassostrea gigas*). Int J Syst Evol Microbiol. (2005) 55:2251–5. 10.1099/ijs.0.63666-0 16280478

[68] Olszak T An D Zeissig S Vera MP Richter J Franke A. Microbial exposure during early life has persistent effects on natural killer T cell function. Science. (2012) 336:489–93. 10.1126/science.1219328 22442383 PMC3437652

[69] De Ridder C Foret TW Non-parasitc symbioses between echinoderms and bacteria. In: Lawrence JM Jangoux M, editors. Echinoderm Studies. Lisse: A. A. Balkema Publishers (2001). p. 111–69.

[70] Cameron RA Holland ND. Electron microscopy of extracellular materials during the development of a sea star, *Patiria miniata* (Echinodermata: Asteroidea). Cell Tissue Res. (1983) 234:193–200. 10.1007/BF00217412 6640617

[71] Cameron RA Holland ND. Demonstration of the granular layer and the fate of the hyaline layer during the development of a sea urchin (*Lytechnius variegatus*). Cell Tissue Res. (1985) 239:455–8. 10.1007/BF00218028 3978700

[72] Walker CW Lesser MP Nutrition and development of brooded embryos in the brittlestar *Amphipholis squamata*: do endosymbiotic bacteria play a role? Mar Biol. (1989) 103:519–30. 10.1007/BF00399584

[73] Cerra A Byrne M Hoegh-Guldberg O Development of the hyaline layer around the planktonic embryos and larvae of the asteroid *Patiriella calcar* and the presence of associated bacteria. Invertebr Reprod Dev. (1997) 31:337–43. 10.1080/07924259.1997.9672594

[74] Manahan DT Davis JP Stephens GC. Bacteria-free sea urchin larvae: selective uptake of neutral amino acids from seawater. Science. (1983) 220:204–6. 10.1126/science.220.4593.204 17795831

[75] Hobbie JE Daley RJ Jasper S. Use of nuclepore filters for counting bacteria by fluorescence microscopy. Appl Environ Microbiol. (1977) 33:1225–8. 327932 10.1128/aem.33.5.1225-1228.1977 PMC170856

[76] Joint I Muhling M Querellou J. Culturing marine bacteria - an essential prerequisite for biodiscovery. Microbiol Biotechnol. (2010) 3:564–75. 10.1111/j.1751-7915.2010.00188.x 21255353 PMC3815769

[77] Bonnell BS Keller SH Vacquier VD Chandler DE. The sea urchin egg jelly coat consists of globular glycoproteins bound to a fibrous fucan superstructure. Dev Biol. (1994) 162:313–24. 10.1006/dbio.1994.1088 8125196

[78] Majeske AJ Oleksyk TK Smith LC. The Sp185/333 immune response genes and proteins are expressed in cells dispersed within all major organs of the adult purple sea urchin. Innate Immun. (2013) 19:569–87. 10.1177/1753425912473850 23405032

[79] Li C Blencke HM Haug T Jorgensen O Stensvag K. Expression of antimicrobial peptides in coelomocytes and embryos of the green sea urchin (*Strongylocentrotus droebachiensis*). Dev Comp Immunol. (2014) 43:106–13. 10.1016/j.dci.2013.10.013 24239709

[80] Stumpp M Hu M Casties I Saborowski R Bleich M Melzner F Digestion in sea urchin larvae impaired under ocean acidification. Nat Clim Change. (2013) 3:1044–9. 10.1038/nclimate2028

[81] Vijayan N Lema KA Nedved BT Hadfield MG Microbiomes of the polychaete *Hydroides elegans* (Polychaeta: Serpulidae) across its life-history stages. Mar Biol. (2019) 166:19 10.1007/s00227-019-3465-9

[82] Carrier TJ Macrander J Reitzel AM A microbial perspective on the life-history evolution of marine invertebrate larvae: If, where and when to feed. Mar Ecol. (2018) 39:e12490 10.1111/maec.12490

[83] Adams DK Sewell MA Angerer RC Angerer LM. Rapid adaptation to food availability by a dopamine-mediated morphogenetic response. Nat Commun. (2011) 2:592. 10.1038/ncomms1603 22186888 PMC3992878

[84] Barnhill AE Brewer MT Carlson SA. Adverse effects of antimicrobials via predictable or idiosyncratic inhibition of host mitochondrial components. Antimicrob Agents Chemother. (2012) 56:4046–51. 10.1128/AAC.00678-12 22615289 PMC3421593

[85] Franks RR Hough-Evans BR Britten RJ Davidson EH. Direct introduction of cloned DNA into the sea urchin zygote nucleus, and fate of injected DNA. Development. (1988) 102:287–99. 3416776 10.1242/dev.102.2.287

[86] Brown SP Cornforth DM Mideo N. Evolution of virulence in opportunistic pathogens: generalism, plasticity, and control. Trends Microbiol. (2012) 20:336–42. 10.1016/j.tim.2012.04.005 22564248 PMC3491314

