# Supplementary material for: Bacterial Exposure Mediates Developmental Plasticity and Resistance to Lethal Vibrio lentus Infection in Purple Sea Urchin (Strongylocentrotus purpuratus) Larvae
Source: Front Immunol. 2020 Jan 14;10:3014. doi: 10.3389/fimmu.2019.03014 (PMC6971090; doi:10.3389/fimmu.2019.03014)
Supplement: Supplementary file 1 [file Data_Sheet_1.PDF]

Supplementary Note 1: QIIME bacterial community profiling procedure

Starting with raw read files from MiSeq:

1. Pair forward and reverse files using PEAR (pear-0.9.10-bin-64)
2. Trim paired files using Trimmomatic (trimmomatic-0.36.jar)
3. Convert paired and trimmed .fastq to .fasta using the custom code: cat [input .fastq] | paste – - - - | cut -f 1,2 | sed 's/^/>/'| tr "\t" "\n" >[output .fasta]
4. Validate tab-delimited mapping file using "validate_mapping_file.py"
5. Generate meta-.fasta files using "add_qiime_labels.py"
6. Detect chimeras from meta-.fasta (called, "combined_seqs.fna")
7. Filter chimeras using "filter_fasta.py"
8. Pick OTUs using "pick_open_reference_otus.py"
9. Filter OTUs with >10 reads using "filter_otus_from_otu_table.py"
10. Filter ‘o__Cryptophyta’ using "filter_taxa_from_otu_table.py"
11. Determine rarefaction depth using "biom summarize-table" and summarized with “alpha_rarefaction.py”
12. Filtered .biom table was rarified using “single_rarefaction.py”
13. Filtered .biom table was split using "split_otu_table.py" to test specific hypotheses
14. Alpha diversity was compared using “alpha_diversity.py”
15. Beta diversity was compared using "jackknifed_beta_diversity.py" and 2D PCoA plots of unweighted and weighted UniFrac values were generated using “make_2d_plots.py”
16. Statistical comparisons of unweighted and weighted UniFrac were performed with “compare_categories.py” using the ‘--method’ “anosim”
17. Taxonomic plots were generated using "summarize_taxa_through_plots.py"
18. OTUs specific to field and laboratory were determined using “shared_phylotypes.py”
