# Supplementary material for: Bacterial Exposure Mediates Developmental Plasticity and Resistance to Lethal Vibrio lentus Infection in Purple Sea Urchin (Strongylocentrotus purpuratus) Larvae
Source: Front Immunol. 2020 Jan 14;10:3014. doi: 10.3389/fimmu.2019.03014 (PMC6971090; doi:10.3389/fimmu.2019.03014)
Supplement: Supplementary file 2 [file Data_Sheet_2.pdf]

Supplementary Table 1: List of genus level OTUs associated with *S. purpuratus* across developmental stages and sites.

Numbers indicate relative proportions (%) of the community represented at each time point. Values < 0.01 are rounded to zero.

					Field			Laboratory		
**Phylum**	**Class**	**Order**	**Family**	**Genus**	**0 dpf**	**4 dpf**	**11 dpf**	**0 dpf**	**4 dpf**	**11 dpf**
Acidobacteria	-	-	-	-	0.00	0.00	0.00	0.00	0.00	0.00
	AT-s2-57	-	-	-	0.00	0.00	0.00	0.00	0.00	0.00
	AT-s54	-	-	-	0.00	0.00	0.00	0.00	0.00	0.00
	Acidobacteria-6	BPC015	-	-	0.00	0.00	0.00	0.00	0.00	0.00
	DA052	Ellin6513	-	-	0.00	0.00	0.00	0.00	0.00	0.00
	Holophagae	Holophagales	-	-	0.00	0.00	0.00	0.00	0.00	0.00
	RB25	-	-	-	0.00	0.00	0.00	0.00	0.00	0.00
	Solibacteres	Solibacterales	Solibacteraceae	*Ca. Solibacter*	0.00	0.00	0.00	0.00	0.00	0.00
	Sva0725	Sva0725	-	-	0.00	0.00	0.00	0.00	0.00	0.00
	Chloracidobacteria	RB41	Ellin6075	-	0.00	0.00	0.00	0.00	0.00	0.00
Actinobacteria	Other	Other	Other	Other	0.00	0.00	0.00	0.00	0.00	0.00
	Acidimicrobiia	Acidimicrobiales	Other	Other	0.00	0.00	0.00	0.00	0.00	0.00
				-	0.00	0.00	0.00	0.00	0.00	0.00
			C111	-	0.02	0.08	0.02	0.00	0.00	0.13
			JdFBGBact	-	0.00	0.00	0.00	0.00	0.00	0.00
			OCS155	-	0.00	0.10	0.00	0.00	0.00	0.33
			SC3-41	-	0.00	0.08	0.04	0.00	0.00	0.03
			TK06	-	0.00	0.00	0.00	0.00	0.00	0.00
			ZA3409c	-	0.00	0.00	0.00	0.00	0.00	0.00
			koll13	-	0.00	0.00	0.00	0.00	0.00	0.00
			ntu14	-	0.00	0.00	0.00	0.00	0.00	0.00
			wb1_P06	-	0.02	0.00	0.00	0.00	0.00	0.03
	Actinobacteria	Actinomycetales	Other	Other	0.00	0.00	0.00	0.00	0.00	0.00
				-	0.00	0.00	0.00	0.00	0.00	0.00
			Cellulomonadaceae	*Demequina*	0.00	0.00	0.00	0.00	0.00	0.00
			Corynebacteriaceae	*Corynebacterium*	0.00	0.00	0.02	0.00	0.03	0.00
			Dermacoccaceae	*Dermacoccus*	0.00	0.00	0.00	0.00	0.00	0.00
			Gordoniaceae	*Gordonia*	0.00	0.00	0.00	0.00	0.00	0.00
			Intrasporangiaceae	-	0.00	0.00	0.00	0.00	0.00	0.00
			Kineosporiaceae	-	0.00	0.00	0.00	0.00	0.00	0.00
			Microbacteriaceae	Other	0.00	0.00	0.00	0.00	0.00	0.00
				-	0.00	0.00	0.00	0.00	0.00	0.10
				Agrococcus	0.00	0.00	0.00	0.00	0.00	0.03
				Ca. Aquiluna	0.00	0.00	0.00	0.00	0.00	0.00
				Microbacterium	0.00	0.00	0.00	0.00	0.00	0.00
				Yonghaparkia	0.00	0.00	0.00	0.00	0.00	0.00
			Micrococcaceae	Other	0.00	0.00	0.00	0.00	0.00	0.00
				Kocuria	0.00	0.00	0.00	0.00	0.00	0.00
				Microbispora	0.00	0.00	0.00	0.00	0.00	0.00
				Micrococcus	0.00	0.00	0.00	0.00	0.00	0.00
			Micromonosporaceae	-	0.00	0.00	0.00	0.00	0.00	0.00
			Mycobacteriaceae	*Mycobacterium*	0.00	0.00	0.00	0.00	0.00	0.00
			Nocardiaceae	Other	0.00	0.00	0.00	0.00	0.00	0.00

				Rhodococcus	0.00	0.00	0.00	0.00	0.00	0.10
			Nocardioidaceae	Other	0.00	0.00	0.00	0.00	0.00	0.00
				-	0.00	0.00	0.00	0.00	0.00	0.00
				Aeromicrobium	0.00	0.00	0.00	0.00	0.00	0.00
				Friedmanniella	0.00	0.00	0.00	0.00	0.00	0.00
				Nocardioides	0.00	0.00	0.02	0.00	0.00	0.00
			Propionibacteriaceae	*Propionibacterium*	0.02	0.00	0.00	0.07	0.00	0.00
			Tsukamurellaceae	Other	0.00	0.00	0.00	0.00	0.00	0.00
	Nitriliruptoria	Euzebyales	Euzebyaceae	*Euzebya*	0.00	0.00	0.00	0.00	0.00	0.03
	Thermoleophilia	Gaiellales	-	-	0.00	0.00	0.00	0.00	0.00	0.00
			AK1AB1_02E	-	0.00	0.00	0.00	0.00	0.00	0.00
Bacteroidetes	Other	Other	Other	Other	0.00	0.00	0.00	0.00	0.00	0.00
	BME43	-		-	0.02	0.00	0.06	0.00	0.00	0.00
	Bacteroidia	Bacteroidales	Other	Other	0.00	0.00	0.00	0.00	0.00	0.00
				-	0.04	0.06	0.06	0.00	0.00	0.03
			Marinilabiaceae	-	0.00	0.00	0.00	0.00	0.00	0.00
			SB-1	-	0.00	0.00	0.00	0.00	0.00	0.00
			VC21_Bac22	-	0.00	0.00	0.00	0.00	0.00	0.00
	Cytophagia	Cytophagales	Other	Other	0.00	0.00	0.00	0.00	0.00	0.00
			Cyclobacteriaceae	-	0.00	0.00	0.00	0.00	0.00	0.03
			Cytophagaceae	-	0.00	0.00	0.00	0.00	0.00	0.00
				Leadbetterella	0.00	0.00	0.00	0.00	0.00	0.00
				Microscilla	0.00	0.00	0.00	0.00	0.00	0.00
				Persicitalea	0.00	0.08	0.00	0.00	0.00	0.00
				Pontibacter	0.00	0.00	0.00	0.00	0.00	0.00
			Flammeovirgaceae	Other	0.00	0.00	0.00	0.00	0.00	0.00
				-	0.06	0.12	0.08	0.00	0.00	0.10
				Flammeovirga	0.00	0.00	0.00	0.00	0.00	0.00
				Flexibacter	0.00	0.00	0.06	0.00	0.00	0.00
				Fulvivirga	0.00	0.00	0.00	0.00	0.00	0.00
				JTB248	0.00	0.02	0.16	0.00	0.00	0.30
				Reichenbachiella	0.00	0.04	0.04	0.00	0.00	0.00
				Roseivirga	0.00	0.02	0.00	0.00	0.00	0.07
			Amoebophilaceae	-	0.00	0.00	0.00	0.00	0.00	0.00
				Ca. Cardinium	0.00	0.00	0.00	0.00	0.00	0.00
				Ucs1325	0.00	0.00	0.00	0.00	0.00	0.00
	Flavobacteriia	-	-	-	0.00	0.00	0.00	0.00	0.00	0.00
		Flavobacteriales	Other	Other	0.00	0.00	0.00	0.00	0.00	0.00
				-	0.10	0.34	0.14	0.00	0.00	0.33
			Cryomorphaceae	Other	0.00	0.00	0.00	0.00	0.00	0.00
				-	0.14	0.62	0.20	0.03	0.00	0.80
				Crocinitomix	0.02	0.10	0.08	0.00	0.00	0.03
				Cryomorpha	0.00	0.00	0.00	0.00	0.00	0.00
				Fluviicola	0.00	0.36	0.04	0.00	0.00	0.30
				Owenweeksia	0.00	0.00	0.00	0.00	0.00	0.00
			Flavobacteriaceae	Other	0.04	0.08	0.10	0.00	0.00	0.07
				-	0.48	2.30	2.46	0.10	0.13	3.20
				Aequorivita	0.00	0.00	0.00	0.00	0.00	0.00
				Aquimarina	0.00	0.00	0.00	0.00	0.00	0.00

				Bizionia	0.00	0.00	0.00	0.00	0.00	0.00
				Flavobacterium	0.12	0.38	0.50	0.00	0.00	0.60
				Gillisia	0.00	0.00	0.00	0.00	0.00	0.00
				Gramella	0.00	0.00	0.00	0.00	0.00	0.00
				Kordia	0.00	0.04	0.02	0.00	0.00	0.00
				Leeuwenhoekiella	0.00	0.36	0.00	0.00	0.00	0.00
				Lutimonas	0.00	0.00	0.02	0.00	0.00	0.00
				Maribacter	0.00	0.00	0.02	0.00	0.00	0.47
				Mesonia	0.00	0.04	0.00	0.00	0.00	0.00
				Muricauda	0.00	0.00	0.00	0.00	0.00	0.00
				Olleya	0.00	0.00	0.42	0.00	0.00	0.13
				Polaribacter	0.00	0.86	0.20	0.00	0.00	0.53
				Salegentibacter	0.00	0.00	0.00	0.00	0.00	0.00
				Sediminicola	0.04	0.56	0.08	0.00	0.00	0.37
				Tenacibaculum	0.06	0.48	0.10	0.00	0.00	0.37
				Ulvibacter	0.00	0.00	0.02	0.00	0.00	0.00
				Winogradskyella	0.00	0.00	0.08	0.00	0.00	0.63
			NS9	-	0.02	0.14	0.04	0.00	0.00	0.50
			Weeksellaceae	-	0.00	0.00	0.00	0.00	0.00	0.00
				Chryseobacterium	0.04	0.06	0.02	0.07	0.10	0.03
				Cloacibacterium	0.00	0.00	0.00	0.00	0.00	0.00
	Sphingobacteriia	Sphingobacteriales	-	-	0.00	0.00	0.00	0.00	0.00	0.00
			NS11-12	-	0.00	0.06	0.00	0.00	0.00	0.07
			Sphingobacteriaceae		0.00	0.00	0.00	0.00	0.00	0.00
				Sphingobacterium	0.00	0.00	0.00	0.00	0.00	0.00
	Rhodothermi	Rhodothermales	Rhodothermaceae	-	0.00	0.02	0.00	0.00	0.00	0.00
				Rubricoccus	0.00	0.00	0.00	0.00	0.00	0.00
			Balneolaceae	*Balneola*	0.00	0.00	0.04	0.00	0.00	0.00
				KSA1	0.00	0.00	0.00	0.00	0.00	0.00
	Saprospirae	Saprospirales	-	-	0.00	0.04	0.08	0.00	0.00	0.03
			Chitinophagaceae	Other	0.00	0.00	0.00	0.00	0.00	0.00
				-	0.00	0.00	0.00	0.00	0.00	0.00
				Flavihumibacter	0.00	0.00	0.00	0.00	0.00	0.00
				Sediminibacterium	0.00	0.00	0.00	0.00	0.00	0.00
			Saprospiraceae	Other	0.00	0.00	0.02	0.00	0.00	0.00
				-	0.22	1.06	0.56	0.00	0.00	0.47
				Lewinella	0.00	0.00	0.02	0.00	0.00	0.00
				Saprospira	0.00	0.06	0.00	0.00	0.00	0.00
Caldithrix	Caldithrixae	Caldithrixales	BA059		0.00	0.00	0.00	0.00	0.00	0.00
	Caldithrixae	Caldithrixales	Caldithrixaceae		0.00	0.00	0.00	0.00	0.00	0.00
Chlamydiae	Chlamydiia	Chlamydiales	Other	Other	0.00	0.00	0.00	0.00	0.00	0.00
				-	0.00	0.00	0.00	0.00	0.00	0.00
			Criblamydiaceae	-	0.00	0.00	0.00	0.00	0.00	0.00
			Parachlamydiaceae	Other	0.14	0.10	0.08	0.20	0.20	0.17
				Ca. Protochlamydia	0.00	0.00	0.00	0.00	0.00	0.00
				Parachlamydia	0.00	0.00	0.00	0.00	0.00	0.00
			Simkaniaceae	Other	0.00	0.00	0.00	0.00	0.00	0.03
Chlorobi	Ignavibacteria	Ignavibacteriales	Ignavibacteriaceae	-	0.00	0.00	0.00	0.00	0.00	0.00
Chloroflexi	Anaerolineae	-	-	-	0.00	0.00	0.00	0.00	0.00	0.00

		Ardenscatenales	Ardenscatenaceae	*Ardenscatena*	0.00	0.02	0.30	0.00	0.00	0.03
		Caldilineales	Caldilineaceae	-	0.00	0.00	0.00	0.00	0.00	0.00
		DRC31	-	-	0.00	0.00	0.00	0.00	0.00	0.00
		GCA004	-	-	0.00	0.00	0.00	0.00	0.00	0.00
		S0208	-	-	0.00	0.00	0.00	0.00	0.00	0.00
		SB-34	-	-	0.00	0.00	0.00	0.00	0.00	0.00
		SBR1031	A4b	-	0.00	0.00	0.00	0.00	0.00	0.00
	Ellin6529	-	-	-	0.00	0.00	0.00	0.00	0.00	0.00
	Ktedonobacteria	Thermogemmatisporales	Thermogemmatisporaceae	-	0.00	0.00	0.00	0.00	0.00	0.00
	SAR202	-	-	-	0.00	0.00	0.00	0.00	0.00	0.00
	TK17	-	-	-	0.00	0.00	0.00	0.00	0.00	0.00
Cyanobacteria	Other	Other	Other	Other	0.00	0.00	0.02	0.00	0.00	0.00
				-	0.00	0.00	0.00	0.00	0.00	0.00
	4C0d-2	MLE1-12	-	-	0.00	0.00	0.00	0.00	0.00	0.03
		SM1D11	-	-	0.00	0.00	0.00	0.00	0.00	0.00
		SM2F09	-	-	0.00	0.00	0.00	0.00	0.00	0.00
		YS2	-	-	0.00	0.00	0.00	0.00	0.00	0.00
	Chloroplast	Other	Other	Other	0.00	0.02	0.02	0.00	0.00	0.00
				-	0.02	0.00	0.00	0.00	0.00	0.00
		Chlorophyta	-	-	0.00	0.00	0.06	0.00	0.00	0.63
			Chlamydomonadaceae	-	0.00	0.00	0.00	0.00	0.00	0.00
			Mamiellaceae	Other	0.00	0.00	0.00	0.00	0.00	0.00
				-	0.00	0.00	0.00	0.00	0.00	0.00
		Haptophyceae	-	-	0.02	0.00	0.00	0.00	0.00	0.00
		Rhodophyta	-	-	0.00	0.00	0.00	0.00	0.00	0.00
		Stramenopiles	-	-	1.80	4.56	8.40	0.00	0.00	2.13
		Streptophyta	-	-	0.02	0.00	0.02	0.03	0.07	0.00
		UA01	-	-	0.02	0.00	0.00	0.00	0.00	0.00
	ML635J-21	-	-	-	0.00	0.00	0.00	0.00	0.00	0.00
	Oscillatoriophycideae	Other	Other	Other	0.00	0.00	0.00	0.00	0.00	0.00
		Chroococcales	Other	Other	0.00	0.00	0.00	0.00	0.00	0.00
			Xenococcaceae	-	0.00	0.00	0.00	0.00	0.00	0.00
	Synechococcophycideae	Other	Other	Other	0.00	0.00	0.00	0.00	0.00	0.00
		Pseudanabaenales	Other	Other	0.00	0.00	0.00	0.00	0.00	0.00
			Pseudanabaenaceae	Other	0.00	0.00	0.00	0.00	0.00	0.00
				-	0.00	0.00	0.00	0.00	0.00	0.00
				Leptolyngbya	0.00	0.00	0.00	0.00	0.00	0.00
		Synechococcales	Synechococcaceae	*Synechococcus*	0.00	0.00	0.02	0.00	0.00	0.07
Fibrobacteres	Fibrobacteria	Fibrobacterales	-	-	0.00	0.00	0.04	0.00	0.00	0.13
		Ucp1540	-	-	0.00	0.00	0.00	0.00	0.00	0.00
	TG3	TG3-2	-	-	0.00	0.00	0.00	0.00	0.00	0.00
Firmicutes	Other	Other	Other	Other	0.00	0.00	0.00	0.00	0.00	0.00
	Bacilli	Bacillales	Bacillaceae	-	0.00	0.00	0.00	0.00	0.00	0.00
				Bacillus	0.00	0.00	0.00	0.00	0.00	0.00
				Marinococcus	0.00	0.00	0.00	0.00	0.00	0.00
			Paenibacillaceae	*Paenibacillus*	0.00	0.00	0.00	0.00	0.00	0.00
			Planococcaceae	-	0.00	0.00	0.00	0.00	0.00	0.00
				Lysinibacillus	0.00	0.00	0.00	0.03	0.00	0.00
				Planococcus	0.00	0.02	0.00	0.00	0.00	0.00

			Staphylococcaceae	*Staphylococcus*	0.00	0.00	0.00	0.00	0.00	0.00
			Thermoactinomycetaceae	-	0.00	0.00	0.00	0.00	0.00	0.00
			Exiguobacteraceae	*Exiguobacterium*	0.00	0.00	0.00	0.00	0.00	0.00
		Lactobacillales	Aerococcaceae	*Facklamia*	0.00	0.00	0.00	0.00	0.00	0.00
			Carnobacteriaceae	*Carnobacterium*	0.00	0.00	0.00	0.00	0.00	0.00
			Streptococcaceae	*Lactococcus*	0.00	0.00	0.00	0.00	0.00	0.00
				Streptococcus	0.00	0.00	0.00	0.00	0.00	0.00
	Clostridia	Other	Other	Other	0.00	0.00	0.00	0.00	0.00	0.00
		Clostridiales	Other	Other	0.00	0.00	0.00	0.00	0.00	0.00
				-	0.00	0.00	0.00	0.00	0.00	0.00
			Clostridiaceae	Other	0.00	0.00	0.00	0.00	0.00	0.00
				-	0.00	0.00	0.00	0.00	0.00	0.00
				Clostridium	0.00	0.00	0.00	0.00	0.00	0.00
			Lachnospiraceae	Other	0.00	0.00	0.00	0.00	0.00	0.00
				-	0.00	0.00	0.00	0.00	0.00	0.00
			Ruminococcaceae	Other	0.00	0.00	0.00	0.00	0.00	0.00
			Veillonellaceae	*Veillonella*	0.00	0.00	0.00	0.00	0.00	0.00
			Acidaminobacteraceae	*Fusibacter*	0.00	0.00	0.00	0.00	0.00	0.00
				WH1-8	0.00	0.00	0.00	0.00	0.00	0.00
			Tissierellaceae	*Finegoldia*	0.00	0.00	0.00	0.00	0.00	0.00
Fusobacteria	Fusobacteriia	Fusobacteriales	Fusobacteriaceae	*Fusobacterium*	0.00	0.00	0.00	0.00	0.00	0.00
				Propionigenium	0.02	0.00	0.02	0.00	0.00	0.00
				Psychrilyobacter	0.00	0.00	0.00	0.00	0.00	0.00
			Leptotrichiaceae	-	0.00	0.00	0.00	0.00	0.00	0.00
GN02	-	-	-	-	0.00	0.00	0.00	0.00	0.00	0.03
	3BR-5F	-	-	-	0.00	0.00	0.00	0.00	0.00	0.00
	BB34	-	-	-	0.00	0.00	0.00	0.00	0.00	0.00
	BD1-5	-	-	-	0.00	0.00	0.02	0.00	0.00	0.03
	IIB17	-	-	-	0.00	0.00	0.00	0.00	0.00	0.00
Gemmatimonadetes	Gemm-2	-	-	-	0.00	0.00	0.00	0.00	0.00	0.00
H-178	-	-	-	-	0.00	0.00	0.00	0.00	0.00	0.00
Lentisphaerae	Lentisphaeria	Lentisphaerales	-	-	0.00	0.00	0.00	0.00	0.00	0.00
			Arctic95B-10	-	0.00	0.00	0.00	0.00	0.00	0.00
			Lentisphaeraceae	-	0.00	0.00	0.00	0.00	0.00	0.00
				Lentisphaera	0.00	0.00	0.00	0.00	0.00	0.00
		Victivallales	Victivallaceae	-	0.00	0.00	0.00	0.00	0.00	0.00
NKB19	-	-	-	-	0.04	0.00	0.06	0.00	0.00	0.00
	SHAB590	-	-	-	0.00	0.00	0.00	0.00	0.00	0.00
Nitrospirae	Nitrospira	Nitrospirales	Nitrospiraceae		0.00	0.00	0.00	0.00	0.00	0.00
OD1	ABY1	-	-	-	0.00	0.00	0.00	0.00	0.00	0.00
	ZB2	-	-	-	0.00	0.00	0.00	0.00	0.00	0.03
OP11	WCHB1-64	d153	-	-	0.00	0.00	0.00	0.00	0.00	0.00
OP3	PBS-25	-	-	-	0.00	0.00	0.00	0.00	0.00	0.00
	koll11	-	-	-	0.00	0.00	0.00	0.00	0.00	0.00
Planctomycetes	Other	Other	Other	Other	0.00	0.00	0.00	0.00	0.00	0.00
				-	0.00	0.00	0.00	0.00	0.00	0.00
	028H05-P-BN-P5	-	-	-	0.00	0.00	0.00	0.00	0.00	0.00
	BD7-11	-	-	-	0.12	0.06	0.08	0.13	0.17	0.07
	C6	d113	-	-	0.00	0.00	0.00	0.00	0.00	0.00

	OM190	Other	Other	Other	0.00	0.00	0.00	0.00	0.00	0.00
				-	0.00	0.00	0.00	0.00	0.00	0.00
		CL500-15	-	-	0.00	0.00	0.06	0.00	0.00	0.03
		agg27	-	-	0.02	0.02	0.06	0.00	0.00	0.07
	Phycisphaerae	CCM11a	-	-	0.00	0.00	0.00	0.00	0.00	0.00
		Phycisphaerales	-	-	0.02	0.00	0.70	0.00	0.00	0.20
			Phycisphaeraceae	-	0.00	0.00	0.00	0.00	0.00	0.00
		S-70	-	-	0.00	0.00	0.00	0.00	0.00	0.00
		mle1-8	-	-	0.00	0.00	0.00	0.00	0.00	0.00
	Pla3	-	-	-	0.00	0.00	0.00	0.00	0.00	0.00
	Planctomycetia	Other	Other	Other	0.00	0.00	0.00	0.00	0.00	0.00
		Gemmatales	Gemmataceae	-	0.00	0.00	0.00	0.00	0.00	0.00
				Gemmata	0.00	0.00	0.00	0.00	0.00	0.00
		Pirellulales	Pirellulaceae	Other	0.00	0.00	0.00	0.00	0.00	0.00
					0.12	0.22	0.24	0.00	0.00	0.23
				A17	0.00	0.00	0.00	0.00	0.00	0.00
		Planctomycetales	Planctomycetaceae	*Planctomyces*	0.02	0.00	0.06	0.00	0.00	0.27
	vadinHA49	-	-	-	0.00	0.00	0.00	0.00	0.00	0.00
Proteobacteria	Other	Other	Other	Other	0.02	0.08	0.02	0.03	0.07	0.00
				-	0.00	0.00	0.00	0.00	0.00	0.00
	Alphaproteobacteria	Other	Other	Other	0.12	0.10	0.28	0.10	0.17	0.13
				-	0.14	0.36	0.18	0.07	0.07	0.70
		BD7-3	-	-	0.00	0.02	0.10	0.00	0.00	0.17
		Caulobacterales	Caulobacteraceae	Other	0.22	0.16	0.10	0.27	0.27	0.13
				-	8.18	5.70	4.24	10.97	10.07	5.57
				Brevundimonas	0.00	0.00	0.00	0.00	0.00	0.00
				Mycoplana	0.02	0.02	0.00	0.07	0.03	0.03
				Phenylobacterium	0.00	0.00	0.00	0.00	0.07	0.00
		Kiloniellales	Other	Other	0.00	0.00	0.00	0.00	0.00	0.00
				-	0.00	0.04	0.02	0.00	0.00	0.03
			Kiloniellaceae	-	0.00	0.02	0.00	0.00	0.00	0.10
				Thalassospira	0.00	0.00	0.00	0.03	0.00	0.00
		Kordiimonadales	Kordiimonadaceae	-	0.00	0.00	0.04	0.00	0.00	0.03
		Rhizobiales	Other	Other	0.28	0.24	0.34	0.13	0.20	0.10
				-	0.64	0.38	0.32	0.63	0.63	0.60
			Aurantimonadaceae	Other	0.00	0.00	0.00	0.00	0.00	0.00
			Beijerinckiaceae	-	0.00	0.00	0.00	0.00	0.00	0.00
			Bradyrhizobiaceae	Other	0.26	0.24	0.36	0.10	0.17	0.00
				-	10.30	9.90	14.70	5.00	6.90	0.33
				Bosea	0.00	0.00	0.00	0.00	0.03	0.00
				Bradyrhizobium	0.20	0.14	0.14	0.33	0.27	0.13
			Hyphomicrobiaceae	Other	0.00	0.00	0.00	0.00	0.00	0.00
				-	0.00	0.00	0.00	0.00	0.00	0.03
				Devosia	0.00	0.00	0.00	0.00	0.00	0.07
				Hyphomicrobium	0.00	0.00	0.00	0.00	0.00	0.00
				Parvibaculum	0.00	0.00	0.00	0.00	0.00	0.00
				Pedomicrobium	0.00	0.00	0.00	0.00	0.00	0.00
				Rhodoplanes	0.00	0.00	0.00	0.00	0.00	0.00
			Methylobacteriaceae	-	0.00	0.00	0.00	0.00	0.00	0.00

		Methylobacterium	0.10	0.06	0.08	0.13	0.13	0.17
	Phyllobacteriaceae	Other	0.04	0.02	0.00	0.03	0.00	0.03
		-	0.10	0.10	0.18	0.07	0.07	0.27
		Mesorhizobium	0.00	0.00	0.00	0.00	0.00	0.00
		Phyllobacterium	0.00	0.00	0.00	0.00	0.00	0.00
	Rhizobiaceae	Other	0.00	0.02	0.04	0.00	0.03	0.00
		-	0.00	0.00	0.00	0.00	0.00	0.00
		Agrobacterium	0.14	0.10	0.08	0.23	0.20	0.13
		Kaistia	0.00	0.00	0.00	0.00	0.00	0.00
		Rhizobium	0.00	0.00	0.00	0.00	0.00	0.00
		Afifella	0.00	0.00	0.00	0.00	0.00	0.00
	Xanthobacteraceae	*Blastochloris*	0.00	0.00	0.00	0.00	0.00	0.00
Rhodobacterales	Other	Other	0.00	0.00	0.00	0.00	0.00	0.03
	Hyphomonadaceae	Other	0.00	0.00	0.00	0.00	0.00	0.00
		-	0.04	0.14	0.54	0.00	0.00	0.37
		Hyphomonas	0.00	0.00	0.12	0.00	0.10	0.03
		Maricaulis	0.00	0.04	0.02	0.00	0.00	0.03
		Oceanicaulis	0.00	0.00	0.04	0.00	0.00	0.00
	Rhodobacteraceae	Other	0.04	0.24	0.44	0.00	0.00	0.27
		-	0.72	4.14	3.00	0.07	0.13	5.63
		Anaerospora	0.00	0.16	0.08	0.00	0.00	0.03
		Dinoroseobacter	0.00	0.00	0.00	0.00	0.00	0.00
		Loktanella	0.00	0.00	0.02	0.00	0.00	0.00
		Marivita	0.00	0.02	0.32	0.00	0.00	0.07
		Oceanicola	0.00	0.00	0.00	0.00	0.00	0.03
		Octadecabacter	0.18	0.90	0.24	0.00	0.00	0.90
		Paracoccus	0.00	0.40	0.00	0.00	0.00	0.00
		Phaeobacter	0.04	0.54	4.40	0.03	1.30	7.57
		Pseudoruegeria	0.00	0.00	0.00	0.00	0.00	0.03
		Rubellimicrobium	0.00	0.00	0.00	0.00	0.00	0.00
		Ruegeria	0.00	0.00	0.00	0.00	0.00	0.00
		Sulfitobacter	0.00	0.22	0.10	0.00	0.00	0.03
		Tropicibacter	0.00	0.00	0.00	0.00	0.00	0.00
Rhodospirillales	-	-	0.00	0.00	0.00	0.00	0.00	0.00
	Acetobacteraceae	Other	0.00	0.00	0.00	0.00	0.00	0.00
		-	0.00	0.00	0.00	0.00	0.00	0.00
	Rhodospirillaceae	Other	0.00	0.00	0.00	0.00	0.00	0.00
		-	0.10	0.16	0.10	0.17	0.13	0.30
		Inquilinus	0.00	0.00	0.00	0.00	0.00	0.00
		Nisaea	0.00	0.00	0.00	0.00	0.00	0.00
Rickettsiales	Other	Other	0.08	0.00	0.00	0.00	0.03	0.00
		-	0.04	0.04	0.06	0.07	0.07	0.03
	AEGEAN_112	-	0.00	0.00	0.00	0.00	0.00	0.00
	Pelagibacteraceae	-	0.02	0.14	0.08	0.00	0.00	0.97
	Rickettsiaceae	-	0.00	0.02	0.04	0.00	0.00	0.10
	mitochondria	Other	0.00	0.00	0.00	0.00	0.00	0.00
Sphingomonadales	Other	Other	0.18	0.40	0.20	0.10	0.10	0.13
		-	0.08	0.44	0.22	0.00	0.00	0.07
	Erythrobacteraceae	Other	0.00	0.00	0.50	0.00	0.00	0.13

			-	0.08	0.82	0.30	0.00	0.00	0.10
			Lutibacterium	0.00	0.00	0.00	0.00	0.00	0.00
		Sphingomonadaceae	Other	1.06	0.94	0.60	1.17	1.30	0.67
			-	0.12	0.10	0.02	0.10	0.17	0.07
			Blastomonas	0.00	0.00	0.00	0.00	0.00	0.00
			Kaistobacter	0.00	0.00	0.00	0.00	0.00	0.00
			Novosphingobium	0.00	0.00	0.00	0.00	0.00	0.00
			Sphingobium	0.00	0.00	0.00	0.00	0.00	0.00
			Sphingomonas	16.68	10.78	7.34	15.87	15.60	8.53
			Sphingopyxis	0.00	0.00	0.00	0.03	0.00	0.03
Betaproteobacteria	Other	Other	Other	0.00	0.00	0.00	0.00	0.00	0.00
			-	0.00	0.02	0.00	0.00	0.00	0.03
	Burkholderiales	Other	Other	0.00	0.00	0.00	0.00	0.00	0.00
		Alcaligenaceae	*Achromobacter*	0.00	0.00	0.00	0.00	0.00	0.00
		Burkholderiaceae	Other	0.00	0.00	0.00	0.00	0.00	0.00
			Burkholderia	0.00	0.00	0.00	0.00	0.00	0.00
			Salinispora	0.00	0.00	0.00	0.00	0.00	0.00
		Comamonadaceae	Other	0.00	0.00	0.00	0.00	0.00	0.00
			-	0.00	0.02	0.00	0.00	0.00	0.00
			Delftia	0.02	0.00	0.00	0.03	0.00	0.00
			RS62	0.00	0.04	0.00	0.00	0.00	0.07
			Thiomonas	0.00	0.00	0.00	0.00	0.00	0.00
			Variovorax	0.00	0.00	0.00	0.00	0.00	0.00
		Oxalobacteraceae	Other	0.00	0.00	0.00	0.00	0.07	0.00
			-	0.02	0.00	0.00	0.00	0.00	0.00
			Cupriavidus	0.06	0.00	0.00	0.03	0.03	0.03
			Janthinobacterium	0.00	0.00	0.00	0.00	0.00	0.00
			Polynucleobacter	0.00	0.00	0.00	0.00	0.00	0.00
			Ralstonia	0.58	0.42	0.36	1.00	0.83	0.50
	Methylophilales	-	-	0.00	0.00	0.00	0.00	0.00	0.00
		Methylophilaceae	-	0.00	0.12	0.06	0.00	0.00	0.10
			Methylotenera	0.00	0.18	0.20	0.00	0.00	0.00
	Nitrosomonadales	Nitrosomonadaceae	Other	0.00	0.00	0.00	0.00	0.00	0.00
			-	0.00	0.00	0.00	0.00	0.00	0.03
	Procabacteriales	Procabacteriaceae		0.00	0.00	0.00	0.00	0.00	0.00
	Rhodocyclales	Rhodocyclaceae	Other	0.00	0.00	0.00	0.00	0.00	0.00
			-	0.00	0.00	0.00	0.00	0.00	0.00
			Dechloromonas	0.00	0.00	0.00	0.00	0.00	0.00
	Tremblayales	Tremblayaceae	Other	0.00	0.00	0.00	0.00	0.00	0.00
Deltaproteobacteria	Other	Other	Other	0.00	0.00	0.00	0.00	0.00	0.00
			-	0.00	0.00	0.00	0.00	0.00	0.00
	Bdellovibrionales	Bacteriovoracaceae	-	0.00	0.02	0.16	0.00	0.00	0.13
			Bacteriovorax	0.00	0.00	0.08	0.00	0.03	0.03
		Bdellovibrionaceae	*Bdellovibrio*	0.00	0.00	0.02	0.00	0.03	0.00
	Desulfobacterales	Desulfobacteraceae	-	0.00	0.00	0.00	0.00	0.00	0.00
			Desulfococcus	0.00	0.00	0.00	0.00	0.00	0.00
			Desulfofrigus	0.00	0.00	0.00	0.00	0.00	0.00
			Desulfosarcina	0.00	0.00	0.00	0.00	0.00	0.00
		Desulfobulbaceae	-	0.00	0.00	0.00	0.00	0.00	0.00

			Desulfocapsa	0.00	0.00	0.00	0.00	0.00	0.00
			Desulfotalea	0.00	0.00	0.00	0.00	0.00	0.00
		Nitrospinaceae	*Nitrospina*	0.02	0.00	0.02	0.00	0.00	0.10
		Desulfovibrionaceae	-	0.00	0.00	0.00	0.00	0.00	0.00
	Desulfuromonadales	Desulfuromonadaceae	-	0.00	0.00	0.00	0.00	0.00	0.00
		Geobacteraceae	*Geobacter*	0.00	0.00	0.00	0.00	0.00	0.00
	GMD14H09	-	-	0.00	0.00	0.04	0.00	0.00	0.00
	Myxococcales	Other	Other	0.00	0.00	0.00	0.00	0.00	0.00
			-	0.02	0.02	0.02	0.00	0.00	0.03
		0319-6G20	-	0.02	0.00	0.00	0.00	0.03	0.03
		Cystobacterineae	-	0.00	0.00	0.00	0.00	0.00	0.00
		Haliangiaceae	-	0.00	0.00	0.00	0.00	0.00	0.00
		Nannocystaceae	*Nannocystis*	0.00	0.00	0.02	0.00	0.00	0.00
			Plesiocystis	0.00	0.00	0.08	0.00	0.00	0.00
		OM27	-	0.06	0.02	0.04	0.00	0.00	0.03
	NB1-j	Other	Other	0.00	0.00	0.00	0.00	0.00	0.00
			-	0.00	0.00	0.00	0.00	0.00	0.00
		JTB38	-	0.00	0.00	0.00	0.00	0.00	0.00
		NB1-i	-	0.00	0.00	0.00	0.00	0.00	0.00
	PB19	-	-	0.00	0.00	0.00	0.00	0.00	0.00
	Spirobacillales	-	-	0.00	0.02	0.00	0.00	0.00	0.00
	Sva0853	-	-	0.00	0.00	0.00	0.00	0.00	0.03
		JTB36	-	0.00	0.00	0.00	0.00	0.00	0.00
		S25_1238	-	0.00	0.00	0.00	0.00	0.00	0.00
		SAR324	-	0.00	0.00	0.00	0.00	0.00	0.07
	Syntrophobacterales	-	-	0.00	0.00	0.00	0.00	0.00	0.00
		Syntrophobacteraceae	-	0.00	0.00	0.00	0.00	0.00	0.03
	Entotheonellales	Entotheonellaceae	*Ca. Entotheonella*	0.00	0.00	0.00	0.00	0.00	0.00
Epsilonproteobacteria	-	-	-	0.00	0.00	0.00	0.00	0.00	0.00
	Campylobacterales	-	-	0.00	0.00	0.00	0.00	0.00	0.00
		Campylobacteraceae	Other	0.00	0.00	0.00	0.00	0.00	0.00
			Arcobacter	0.22	0.10	0.06	0.00	0.00	0.07
			Sulfurospirillum	0.00	0.00	0.00	0.00	0.00	0.00
		Helicobacteraceae	-	0.00	0.00	0.00	0.00	0.00	0.00
			Sulfurimonas	0.00	0.00	0.00	0.00	0.00	0.00
Gammaproteobacteria	Other	Other	Other	0.40	0.70	0.56	0.40	0.47	0.80
			-	0.16	0.12	0.28	0.07	0.07	0.10
	34P16	-	-	0.00	0.04	0.06	0.00	0.00	0.07
	Aeromonadales	Aeromonadaceae	Other	0.00	0.00	0.00	0.00	0.00	0.03
			-	0.00	0.00	0.02	0.00	0.00	0.00
		Succinivibrionaceae	-	0.00	0.00	0.00	0.00	0.00	0.00
		Other	Other	0.00	0.08	0.06	0.00	0.00	0.03
			-	0.08	0.16	0.16	0.00	0.00	0.07
		Alteromonadaceae	Other	0.00	0.10	0.22	0.00	0.00	0.03
			-	0.54	1.18	2.66	0.00	0.40	0.67
			Alteromonas	0.14	0.56	0.64	0.00	0.13	0.13
			BD2-13	0.00	0.44	0.06	0.00	0.00	0.03
			Ca. Endobugula	0.00	0.00	0.00	0.00	0.00	0.00
			Cellvibrio	0.00	0.00	0.00	0.00	0.00	0.00

		Glaciecola	0.06	0.10	0.08	0.00	0.00	0.07
		HB2-32-21	0.00	0.00	0.00	0.00	0.00	0.03
		HTCC2207	0.06	0.32	0.12	0.00	0.00	0.33
		Marinobacter	0.00	0.02	0.04	0.10	0.03	0.13
		ZD0117	0.00	0.00	0.00	0.00	0.00	0.00
	Colwelliaceae	Other	0.00	0.06	0.14	0.00	0.00	0.03
		-	0.28	0.66	2.20	0.03	0.53	0.27
		Colwellia	0.00	0.00	0.04	0.00	0.00	0.07
		Thalassomonas	0.00	0.00	0.00	0.00	0.00	0.00
	Ferrimonadaceae	*Ferrimonas*	0.00	0.00	0.00	0.03	0.00	0.00
	HTCC2188	-	0.00	0.00	0.00	0.00	0.00	0.00
		HTCC	0.04	0.28	0.14	0.00	0.00	0.20
	Idiomarinaceae	-	0.00	0.00	0.00	0.00	0.00	0.00
		Idiomarina	0.00	0.00	0.00	0.00	0.00	0.00
		Pseudidiomarina	0.00	0.00	0.00	0.00	0.00	0.00
	J115	Other	0.00	0.00	0.02	0.00	0.00	0.00
		-	0.00	0.00	0.06	0.00	0.00	0.07
	Moritellaceae	*Moritella*	0.10	0.16	0.04	0.00	0.00	0.03
	OM60	Other	0.00	0.00	0.00	0.00	0.00	0.00
		-	0.14	0.36	0.34	0.00	0.00	0.33
		Congregibacter	0.00	0.00	0.00	0.00	0.00	0.00
	Psychromonadaceae	*Psychromonas*	0.30	0.50	0.24	0.00	0.00	0.17
	Shewanellaceae	*Shewanella*	0.00	0.18	0.18	0.00	0.00	0.27
	Chromatiaceae	Other	0.00	0.00	0.00	0.00	0.00	0.00
		-	0.00	0.02	0.00	0.00	0.00	0.00
		Rheinheimera	0.00	0.00	0.00	0.00	0.00	0.00
Chromatiales	Other	Other	0.00	0.00	0.00	0.00	0.00	0.00
		-	0.08	0.30	0.18	0.00	0.00	0.07
	Chromatiaceae	-	0.00	0.00	0.00	0.00	0.00	0.00
	Ectothiorhodospiraceae	-	0.00	0.00	0.00	0.00	0.00	0.00
Enterobacteriales	Enterobacteriaceae	Other	0.00	0.02	0.06	0.00	0.00	0.00
		-	0.00	0.00	0.00	0.00	0.00	0.00
		Dickeya	0.00	0.08	0.14	0.00	0.00	0.03
HOC36	-	-	0.00	0.00	0.00	0.00	0.00	0.00
HTCC2188	-	-	0.00	0.00	0.00	0.00	0.00	0.00
	HTCC2089	-	0.00	0.06	0.06	0.00	0.00	0.10
Legionellales	Other	Other	0.00	0.00	0.00	0.00	0.00	0.00
		-	0.00	0.00	0.00	0.00	0.00	0.00
	Coxiellaceae	-	0.00	0.00	0.04	0.00	0.00	0.07
		Aquicella	0.00	0.00	0.00	0.00	0.00	0.00
	Endoecteinascidiaceae	-	0.00	0.00	0.00	0.00	0.00	0.00
	Francisellaceae	-	0.00	0.00	0.00	0.00	0.00	0.03
		Francisella	0.00	0.00	0.00	0.00	0.00	0.00
	Legionellaceae	-	0.00	0.06	0.00	0.03	0.03	0.00
		Legionella	0.00	0.00	0.00	0.00	0.00	0.00
Methylococcales	Other	Other	0.00	0.00	0.00	0.00	0.00	0.00
		-	0.00	0.00	0.00	0.00	0.00	0.00
	Crenotrichaceae	*Crenothrix*	0.00	0.00	0.00	0.00	0.00	0.00
	Methylococcaceae	*Methylomonas*	0.00	0.00	0.00	0.00	0.00	0.00

Oceanospirillales	Other	Other	0.00	0.00	0.20	0.00	0.00	0.17
		-	0.10	0.36	0.18	0.00	0.00	0.67
	Alcanivoracaceae	*Alcanivorax*	0.00	0.00	0.06	0.47	0.40	0.27
	Endozoicimonaceae	-	0.00	0.02	0.00	0.00	0.00	0.03
	Halomonadaceae	Other	0.00	0.00	0.00	0.00	0.00	0.00
		-	0.00	0.00	0.04	0.00	0.00	0.00
		Ca. Portiera	0.26	1.18	0.44	0.07	0.03	1.83
		Haererehalobacter	0.00	0.00	0.00	0.00	0.00	0.00
		Halomonas	0.00	0.04	0.00	0.00	0.03	0.00
	Oceanospirillaceae	Other	0.04	0.60	0.26	0.00	0.00	0.57
		-	0.14	0.52	0.16	0.00	0.00	0.20
		Amphritea	0.00	0.02	0.00	0.00	0.00	0.00
		Marinobacterium	0.00	0.00	0.00	0.00	0.00	0.00
		Marinomonas	0.00	0.08	0.02	0.00	0.00	0.03
		Neptunomonas	0.00	0.00	0.00	0.00	0.00	0.00
		Oceanospirillum	0.00	0.00	0.00	0.00	0.00	0.00
		Oleibacter	0.04	0.20	0.36	0.00	0.00	0.20
		Oleispira	0.02	1.32	0.64	0.00	0.00	1.43
	Oleiphilaceae	-	0.02	0.12	0.14	0.00	0.00	0.07
	SUP05	-	0.00	0.00	0.00	0.00	0.00	0.03
	Saccharospirillaceae	Other	0.00	0.00	0.00	0.00	0.00	0.00
		-	0.00	0.00	0.00	0.00	0.00	0.00
Pseudomonadales	Other	Other	0.00	0.00	0.00	0.00	0.00	0.00
	Moraxellaceae	Other	0.00	0.00	0.00	0.00	0.00	0.00
		-	0.00	0.00	0.00	0.00	0.00	0.00
		Acinetobacter	0.04	0.02	0.00	0.10	0.00	0.00
		Alkanindiges	0.00	0.00	0.00	0.00	0.00	0.00
		Enhydrobacter	0.00	0.00	0.00	0.00	0.00	0.00
		Moraxella	0.02	0.00	0.02	0.03	0.03	0.00
		Perlucidibaca	0.00	0.24	0.80	0.00	0.00	0.00
		Psychrobacter	0.00	0.10	0.02	0.00	0.00	0.00
	Pseudomonadaceae	Other	0.64	0.48	0.34	1.03	0.83	0.47
		-	1.36	1.18	0.74	1.77	1.83	0.97
		Azorhizophilus	0.00	0.00	0.00	0.00	0.00	0.00
		Pseudomonas	29.56	19.02	15.40	38.37	37.90	21.40
Salinisphaerales	-	-	0.00	0.00	0.00	0.00	0.00	0.00
	Salinisphaeraceae	-	0.00	0.00	0.00	0.00	0.00	0.00
		Salinisphaera	0.00	0.00	0.00	0.00	0.00	0.00
Thiohalorhabdales	-	-	0.14	0.14	0.12	0.00	0.00	0.03
	Thiohalorhabdaceae	-	0.00	0.00	0.00	0.00	0.00	0.00
Thiotrichales	Other	Other	0.00	0.00	0.00	0.00	0.00	0.00
		-	0.00	0.00	0.00	0.00	0.00	0.00
	Piscirickettsiaceae	Other	0.00	0.00	0.00	0.00	0.00	0.00
		-	0.08	0.04	0.08	0.00	0.00	0.17
		Methylophaga	0.00	0.38	0.00	0.03	0.03	0.00
		Piscirickettsia	0.00	0.00	0.00	0.00	0.00	0.00
	Thiotrichaceae	*Cocleimonas*	0.00	0.00	0.00	0.00	0.00	0.00
		Leucothrix	0.00	0.00	0.04	0.00	0.00	0.00
		Thiothrix	0.00	0.04	0.02	0.00	0.00	0.00

		Vibrionales	Other	Other	0.00	0.00	0.00	0.00	0.00	0.00
			Pseudoalteromonadaceae	Other	0.00	0.00	0.00	0.00	0.00	0.00
				Pseudoalteromonas	0.04	0.14	0.32	0.00	0.03	0.43
			Vibrionaceae	Other	0.00	0.02	0.00	0.00	0.00	0.00
				-	0.20	0.46	0.34	0.00	0.00	0.20
				Aliivibrio	0.00	0.00	0.00	0.00	0.00	0.00
				Enterovibrio	0.00	0.00	0.00	0.00	0.00	0.00
				Photobacterium	0.00	0.00	0.00	0.00	0.00	0.00
				Vibrio	0.04	0.08	0.06	0.00	0.00	0.03
		Xanthomonadales	Sinobacteraceae	-	0.28	0.20	0.22	0.37	0.33	0.27
				Nevskia	0.00	0.00	0.00	0.00	0.00	0.00
			Xanthomonadaceae	Other	2.42	1.42	1.02	2.70	2.23	1.50
				-	0.24	0.12	0.10	0.23	0.27	0.17
				Dokdonella	0.00	0.00	0.00	0.00	0.00	0.00
				Luteimonas	0.00	0.00	0.00	0.00	0.00	0.00
				Lysobacter	0.00	0.00	0.00	0.00	0.00	0.00
				Pseudoxanthomonas	0.00	0.00	0.00	0.00	0.00	0.00
				Stenotrophomonas	13.68	7.48	5.78	14.87	12.83	8.37
		Marinicellales	Marinicellaceae	-	0.02	0.00	0.04	0.00	0.00	0.00
				Marinicella	0.06	0.06	0.14	0.00	0.00	0.03
	TA18	CV90	-	-	0.00	0.00	0.00	0.00	0.00	0.00
		PHOS-HD29	-	-	0.00	0.00	0.00	0.00	0.00	0.00
SAR406	AB16	Arctic96B-7	A714017	SGSH944	0.00	0.04	0.04	0.00	0.00	0.20
				SargSea-WGS	0.00	0.00	0.00	0.00	0.00	0.00
				ZA3312c	0.00	0.00	0.00	0.00	0.00	0.07
SBR1093	A712011	-	-	-	0.00	0.00	0.00	0.00	0.00	0.00
	EC214	-	-	-	0.00	0.00	0.00	0.00	0.00	0.00
	VHS-B5-50	-	-	-	0.00	0.00	0.00	0.00	0.00	0.03
SR1	-	-	-	-	0.00	0.00	0.00	0.00	0.00	0.00
Spirochaetes	Spirochaetes	Spirochaetales	Spirochaetaceae	*Spirochaeta*	0.00	0.00	0.00	0.00	0.00	0.00
TM6	SBRH58	-	-	-	0.00	0.00	0.00	0.00	0.00	0.00
	SJA-4	-	-	-	0.00	0.00	0.02	0.00	0.00	0.00
		S1198	-	-	0.00	0.00	0.00	0.00	0.00	0.00
TM7	-	-	-	-	0.00	0.00	0.00	0.00	0.00	0.00
	TM7-1	-	-	-	0.00	0.00	0.00	0.00	0.00	0.00
Tenericutes	Mollicutes	Mycoplasmatales	Mycoplasmataceae	*Ca. Hepatoplasma*	0.00	0.00	0.00	0.00	0.00	0.00
				Mycoplasma	0.00	0.00	0.00	0.00	0.00	0.00
Verrucomicrobia	Other	Other	Other	Other	0.00	0.00	0.00	0.00	0.00	0.00
	Opitutae	Other	Other	Other	0.00	0.00	0.00	0.00	0.00	0.00
				-	0.00	0.00	0.00	0.00	0.00	0.00
		Opitutales	Opitutaceae	-	0.00	0.00	0.00	0.00	0.00	0.00
				Opitutus	0.00	0.00	0.00	0.00	0.00	0.00
		Puniceicoccales	Puniceicoccaceae	-	0.00	0.00	0.00	0.00	0.00	0.00
				Coraliomargarita	0.00	0.08	0.12	0.00	0.00	0.07
				MB11C04	0.04	0.18	0.08	0.00	0.00	0.33
		Cerasicoccales	Cerasicoccaceae	-	0.00	0.00	0.00	0.00	0.00	0.00
		Pelagicoccales	Pelagicoccaceae	*Pelagicoccus*	0.00	0.00	0.00	0.00	0.00	0.03
	Verruco-5	LD1-PB3	-	-	0.00	0.00	0.00	0.00	0.00	0.00
			LD1-PA34	-	0.00	0.00	0.00	0.00	0.00	0.00

		MSBL3	-	-	0.00	0.00	0.00	0.00	0.00	0.00
		R76-B128	-	-	0.02	0.00	0.00	0.00	0.00	0.00
		SS1-B-03-39	-	-	0.00	0.00	0.00	0.00	0.00	0.00
		WCHB1-41	-	-	0.00	0.00	0.00	0.00	0.00	0.00
	Verrucomicrobiae	Verrucomicrobiales	Verrucomicrobiaceae	Other	0.00	0.00	0.00	0.00	0.00	0.00
				-	0.12	0.24	0.20	0.00	0.00	0.37
				Akkermansia	0.00	0.00	0.00	0.00	0.00	0.00
				Luteolibacter	0.00	0.00	0.00	0.00	0.00	0.00
				MSBL3	0.00	0.00	0.00	0.00	0.00	0.00
				Persicirhabdus	0.24	0.52	0.52	0.00	0.00	1.13
				Rubritalea	0.00	0.02	1.24	0.00	0.00	0.10
				Verrucomicrobium	0.00	0.00	0.20	0.00	0.00	0.13
	Methylacidiphilae	Methylacidiphilales	LD19	-	0.00	0.00	0.00	0.00	0.00	0.00
	[Pedosphaerae]	Other	Other	Other	0.00	0.00	0.00	0.00	0.00	0.00
				-	0.00	0.10	0.04	0.00	0.00	0.10
		Arctic97B-4	-	-	0.00	0.00	0.00	0.00	0.00	0.00
WPS-2	-	-	-	-	0.00	0.00	0.00	0.00	0.00	0.00
WS3	PRR-12	GN03	KSB4	-	0.00	0.00	0.00	0.00	0.00	0.00
		Sediment-1	-	-	0.00	0.00	0.00	0.00	0.00	0.00
			CV106	-	0.00	0.00	0.00	0.00	0.00	0.00
		wb1_H11		-	0.00	0.00	0.00	0.00	0.00	0.00
WS6	B142	-	-	-	0.00	0.00	0.00	0.00	0.00	0.00
	SC72	-	-	-	0.00	0.00	0.00	0.00	0.00	0.00
ZB3	BS119	-	-	-	0.00	0.00	0.00	0.00	0.00	0.00
Thermi	Deinococci	Deinococcales	Deinococcaceae	*Deinococcus*	0.00	0.00	0.00	0.00	0.00	0.00
			Trueperaceae	-	0.00	0.00	0.00	0.00	0.00	0.00
Other	Other	Other	Other	Other	0.62	0.88	1.10	0.33	0.27	0.97
	Other	Other	Other	Other	0.00	0.00	0.00	0.00	0.00	0.00

Supplementary Table 2: Larval morphometrics principal component analysis.
(A) Total variance explained.

	Initial Eigenvalues			Extraction Sums of Squared Loadings		
**Component**	**Total**	**% of Variance**	**Cumulative %**	**Total**	**% of Variance**	**Cumulative %**
1	3.713	74.261	74.261	3.713	74.261	74.261
2	0.936	18.71	92.971	0.936	18.71	92.971
3	0.224	4.475	97.446			
4	0.092	1.849	99.295			
5	0.035	0.705	100			

(B) Component matrix (2 components extracted).

	Component	
	**1**	**2**
Body length	0.959	0.071
Anterolateral rod length	0.961	−0.120
Postoral rod length	0.950	−0.159
Body rod length	0.357	0.932
Midgut area	0.916	−0.146

<u>Supplementary Table 3</u>: PCR primers used for *Vibrio* multilocus sequence typing.

Target	Primer	Primer sequence	Reference
16S	Eub11f3mx	5'-TGRGTTTGATCMTGGCTYAG-3'	Watanabe et al. 2003
	Eub1511r1mx	5'-TGGHTACCTTGTTACGACTT-3'	
rpoD	70F	5'-ACGACTGACCCGGTACGCATGTAYATGMGNGARATGGGNACNGT-3'	Pascual et al. 2010
	70R	5'-ATAGAAATAACCAGACGTAAGTTNGCYTCNACCATYTCYTTYT-3'	
toxR	toxRs	5'-GANCARGGNTTYGARGTNGAYGAYTC-3'	Pascual et al. 2010
	toxRas	5'-TTDKKTTGNCCNCYNGTVGCDATNAC-3'	

<u>Supplementary Table 4</u>: Multilocus sequence typing results for laboratory and field *Vibrio lentus* strains.

Target	Strain	Top BLAST hit	Query cover (%)	E-value	Identity (%)	Accession #
16S	V1 (laboratory)	Multiple *Splendidus* clade *Vibrios*	100	0.0	100.00	-
	V7 (field)	*Vibrio splendidus* LGP32 chromosome 1	97	0.0	96.46	FM954972.2
rpoD	V1 (laboratory)	*Vibrio lentus* partial *rpoD* gene for RNA polymerase sigma factor RpoD, isolate 3OM12	100	0.0	99.61	LN832741.1
	V7 (field)	*Vibrio lentus* partial *rpoD* gene for RNA polymerase sigma factor RpoD, isolate 3OM12	100	0.0	97.27	LN832741.1
toxR	V1 (laboratory)	*Vibrio lentus* strain CIP 107166 transmembrane regulatory protein (*toxR*) gene, partial cds	100	0.0	97.93	AY751359.1
	V7 (field)	*Vibrio lentus* strain CIP 107166 transmembrane regulatory protein (*toxR*) gene, partial cds	99	0.0	98.26	AY751359.1
