# Supplementary material for: Bacterial Exposure Mediates Developmental Plasticity and Resistance to Lethal Vibrio lentus Infection in Purple Sea Urchin (Strongylocentrotus purpuratus) Larvae
Source: Front Immunol. 2020 Jan 14;10:3014. doi: 10.3389/fimmu.2019.03014 (PMC6971090; doi:10.3389/fimmu.2019.03014)
Supplement: Supplementary file 3 [file Data_Sheet_3.PDF]

	P/S	FSW	AEW
**Replicate 1**	8.19	8.28	8.26
**Replicate 2**	8.19	8.28	8.25

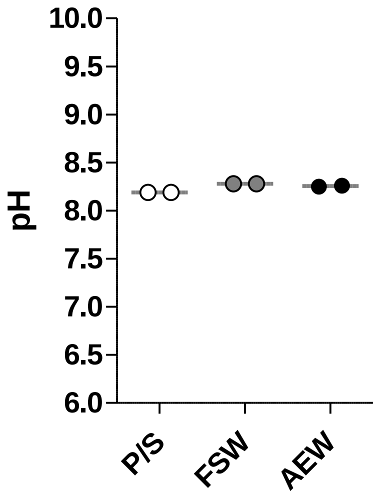

<u>Supplementary Figure 1</u>: Bacterial load-modifying saltwater treatments do not appreciably alter medium pH. P/S and AEW were prepared from two independent batches of FSW and tested for pH after incubation for 2 days at 14°C.

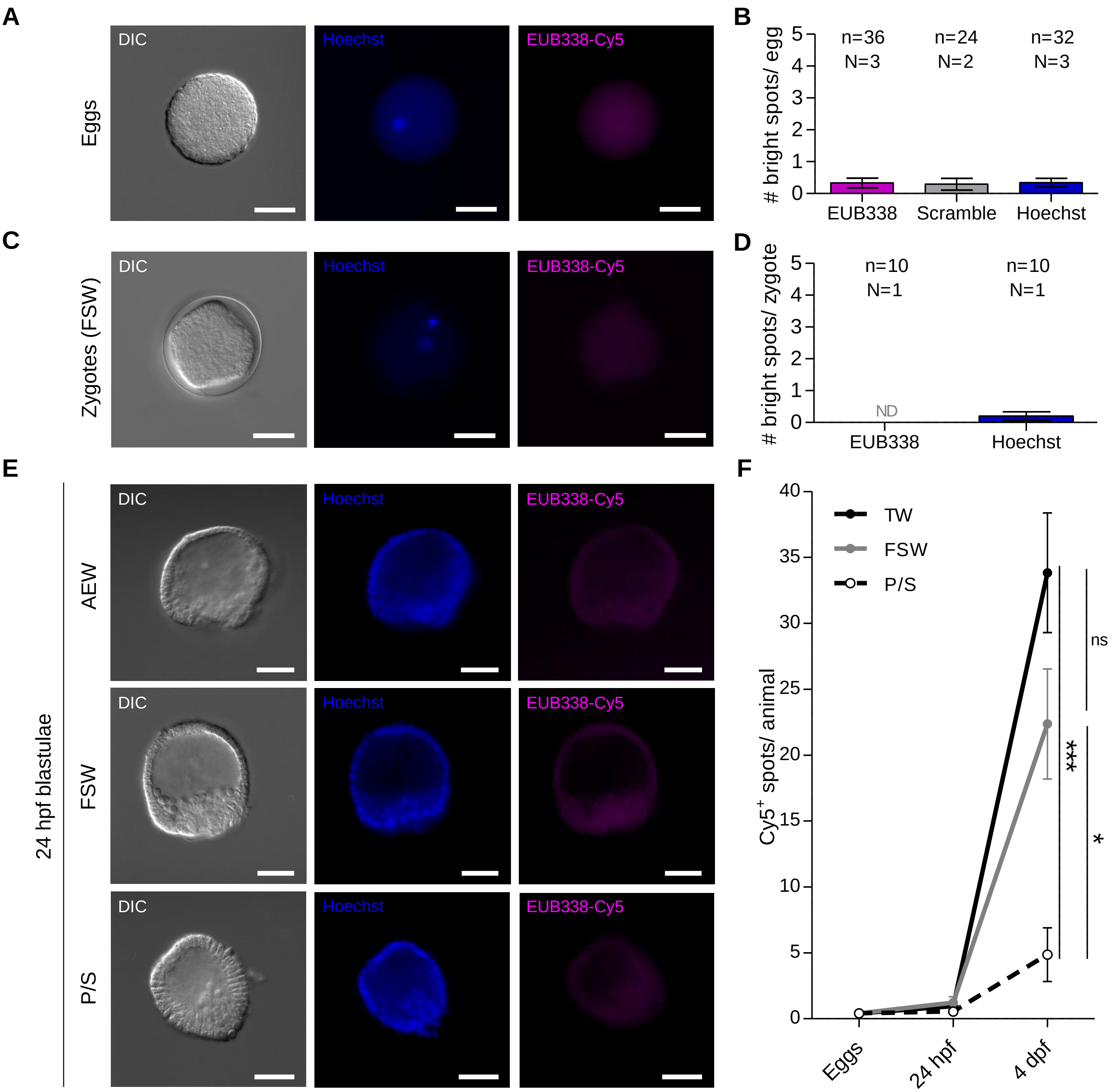

Supplementary Figure 2: Few bacteria are detectable in purple sea urchin eggs, zygotes, or early embryos by 16S fluorescent *in situ* hybridization. Eggs, zygotes, early embryos, and larvae were raised, collected, and fixed as described and stained with the pan-bacterial 16S probe EUB338 conjugated to Cy5 (magenta) with Hoechst counterstain (blue). (**A**) DIC and fluorescent images of eggs collected directly from gonopores. (**B**) Quantification of EUB338-Cy5 and Hoechst staining of eggs, compared to a Cy5-conjugated scramble probe control. (**C,D**) Imaging and quantification of EUB338-stained zygotes. ND, not detected. (**E**) EUB338-stained 24 hpf blastulae raised in P/S, FSW, or TW. (**F**) EUB338 staining quantified for each treatment across development to 4-arm larva stage. All scale bars = 50 μm. **, $p < 0.01$; ***, $p < 0.0001$, 1-way ANOVA with post-hoc Bonferroni tests. Error bars represent mean +/- SEM.

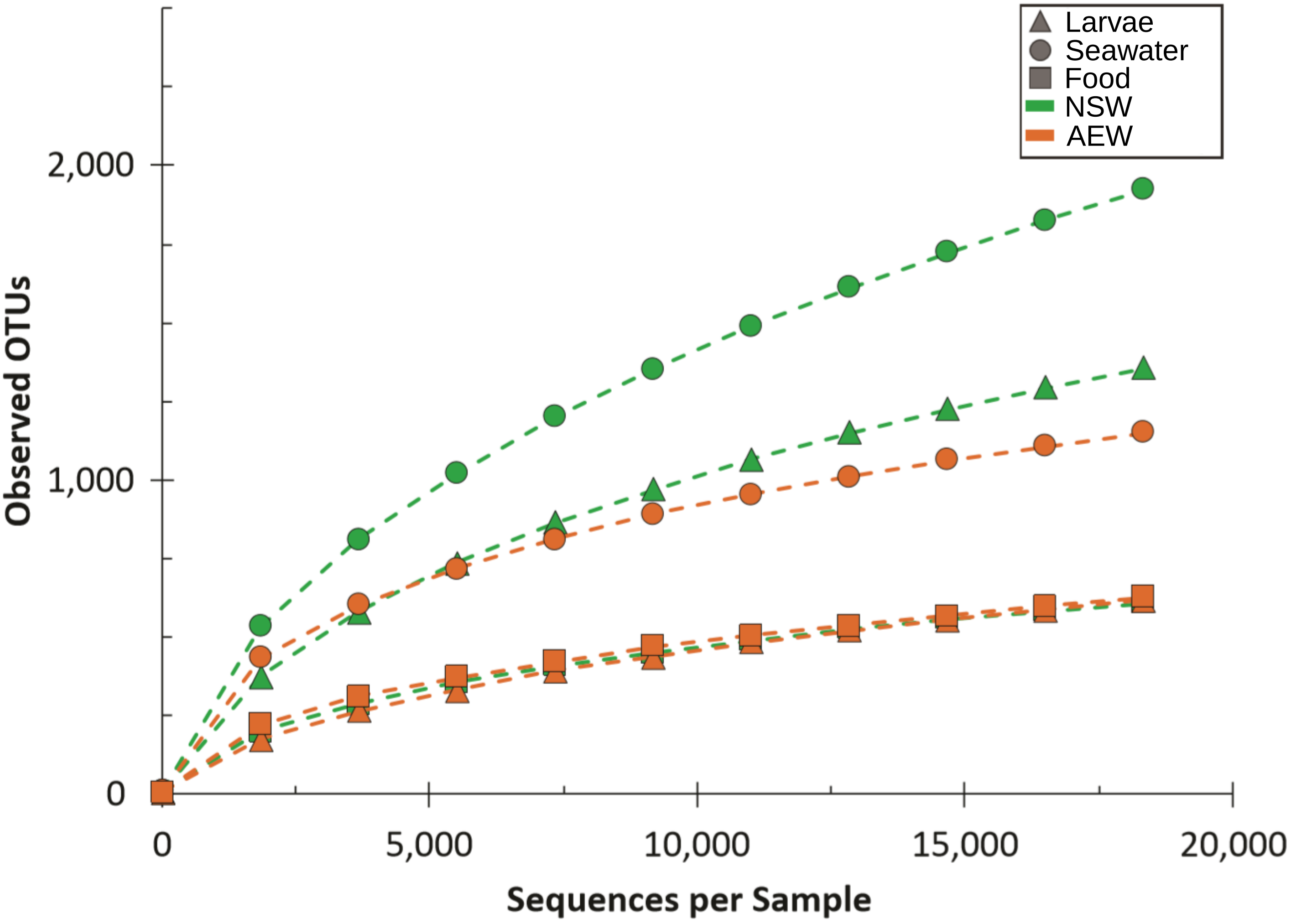

Supplementary Figure 3: Alpha rarefaction curves for larvae, seawater, and algal cultures in the laboratory (AEW) and in natural seawater (NSW). Curves were calculated for 0, 4, and 11 dpf *S. purpuratus* developmental stages (triangles), *Rhodomonas lens* feeding cultures (squares), and environmental culture water microbiota (circles) raised in NSW at Friday Harbor (green) or in the laboratory in AEW (orange) based on a depth of 18, 350 sequences. This depth was subsequently applied to all metagenomic analyses in this study.

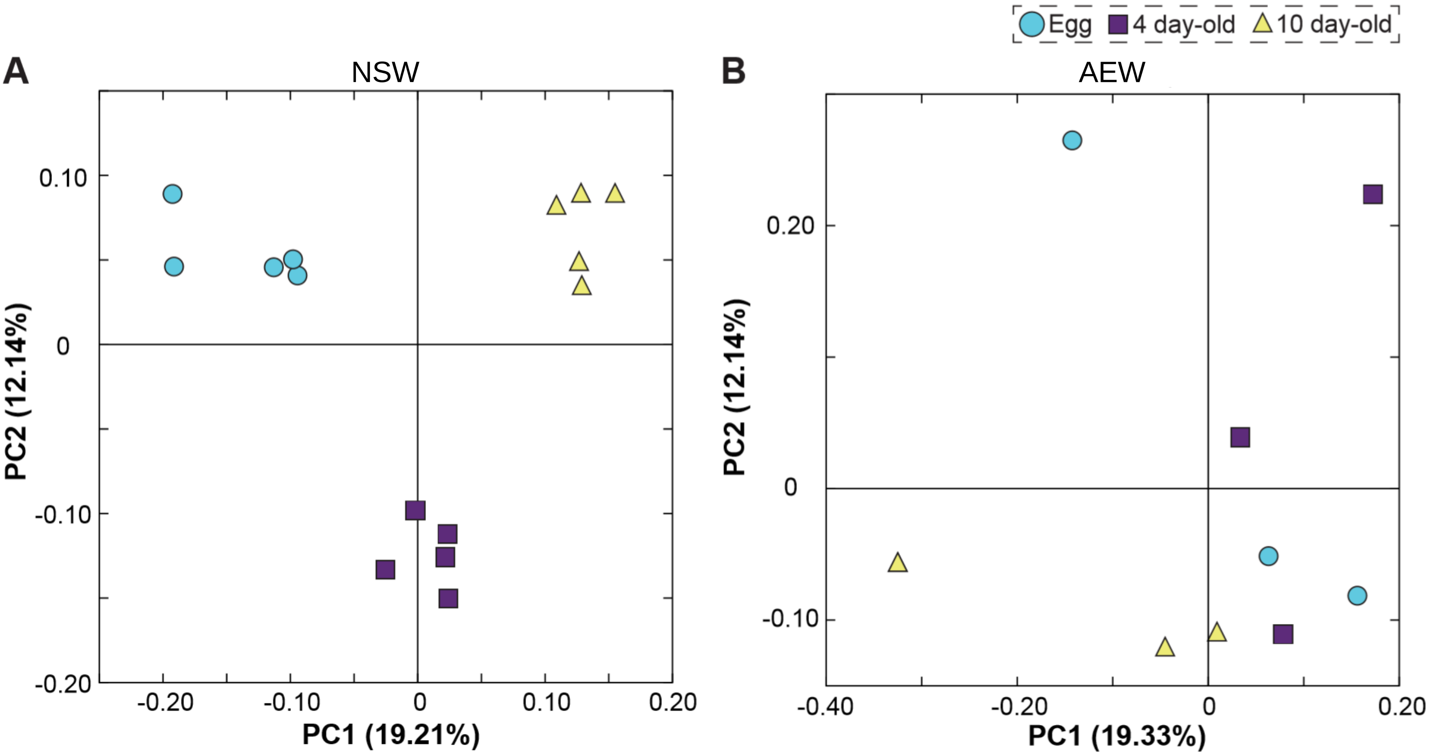

<u>**Supplementary Figure 4**</u>**: Unweighted similarity of *S. purpuratus* larva-associated bacterial communities across developmental stages.** Bacterial community similarity between 0 dpf (turquoise circles), 4 dpf unfed larvae (purple squares), and 10-11 dpf feeding larvae (yellow triangles) was analyzed by principle coordinate analysis. Unweighted UniFrac values are shown for animals raised in natural seawater **(A)** and artificial seawater supplemented with 20% 40 µm-filtered adult-exposed tank water in the laboratory **(B)**.

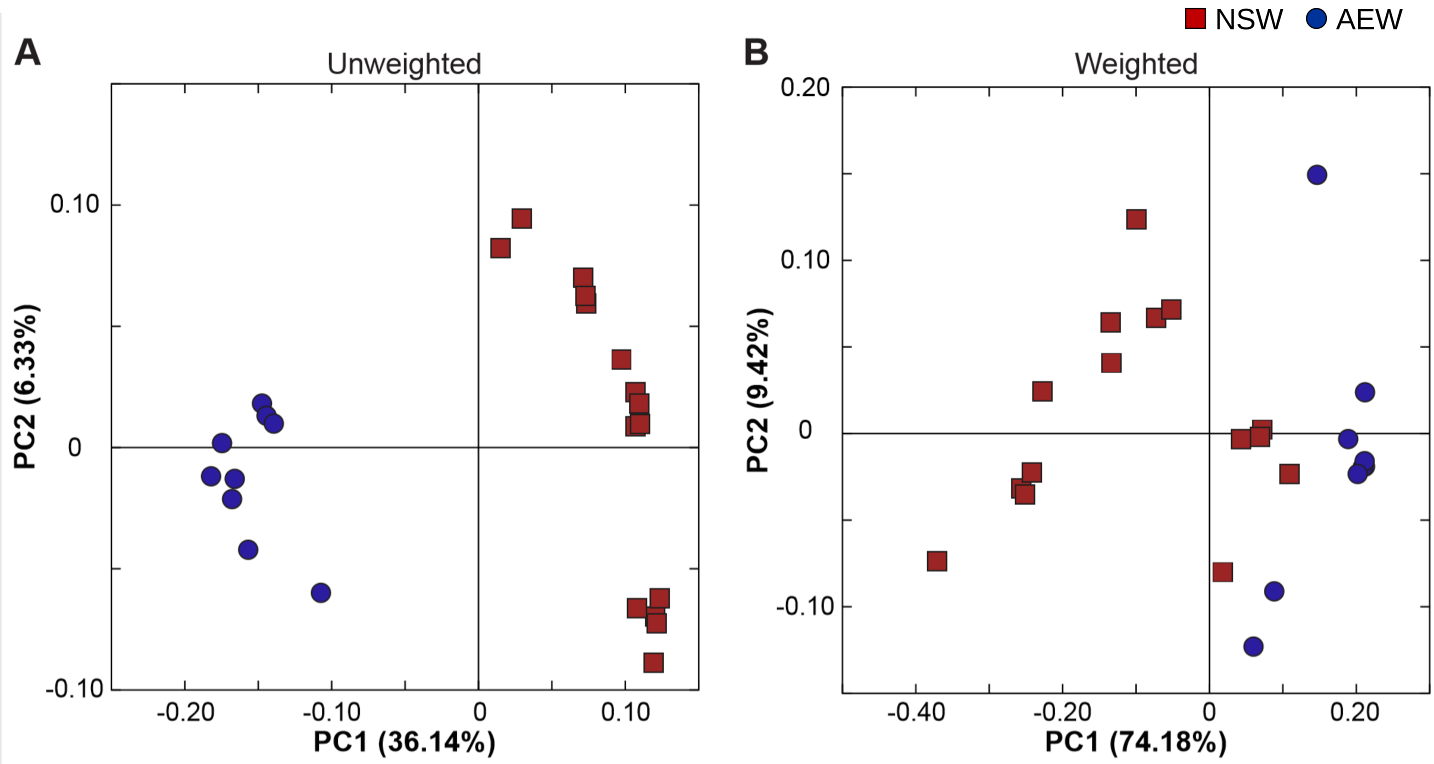

Supplementary Figure 5: Similarity of *S. purpuratus* larva-associated bacterial communities between culture conditions. Bacterial community similarity between animals raised in natural seawater (NSW, red squares) or artificial seawater supplemented with 20% 40 μm-filtered adult-exposed tank water in the laboratory (AEW, blue circles) was analyzed by principle coordinate analysis. Unweighted **(A)** and weighted **(B)** UniFrac results are shown.

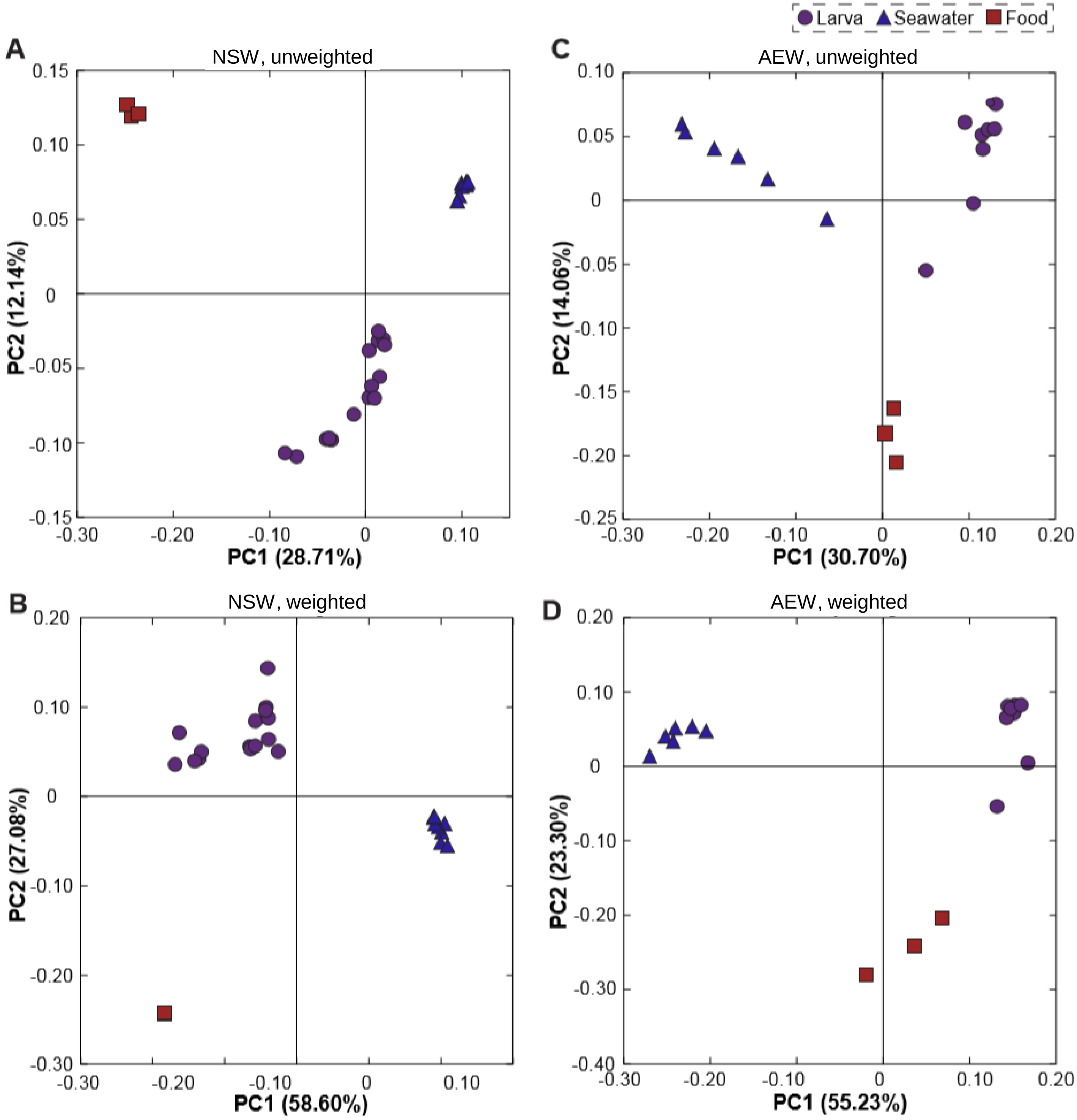

<u>Supplementary Figure 6</u>: **Similarity between associated bacterial communities of *S. purpuratus* developmental stages, algal food cultures, and environment**. Bacterial community similarity among *S. purpuratus* 0 dpf zygotes, 4 dpf unfed larvae, and 11 dpf feeding larvae (purple circles); culture water (blue triangles); and algal feeding cultures (*Rhodomonas lens*, red squares) raised in natural seawater (NSW, left; A, B) or in the laboratory in artificial seawater supplemented with 20% 40 µm-filtered adult-exposed tank water (AEW, right; C, D) was compared by principle coordinate analysis. **(A, C)** Unweighted principle coordinate analyses. **(B, D)** The same analyses weighted by UniFrac.

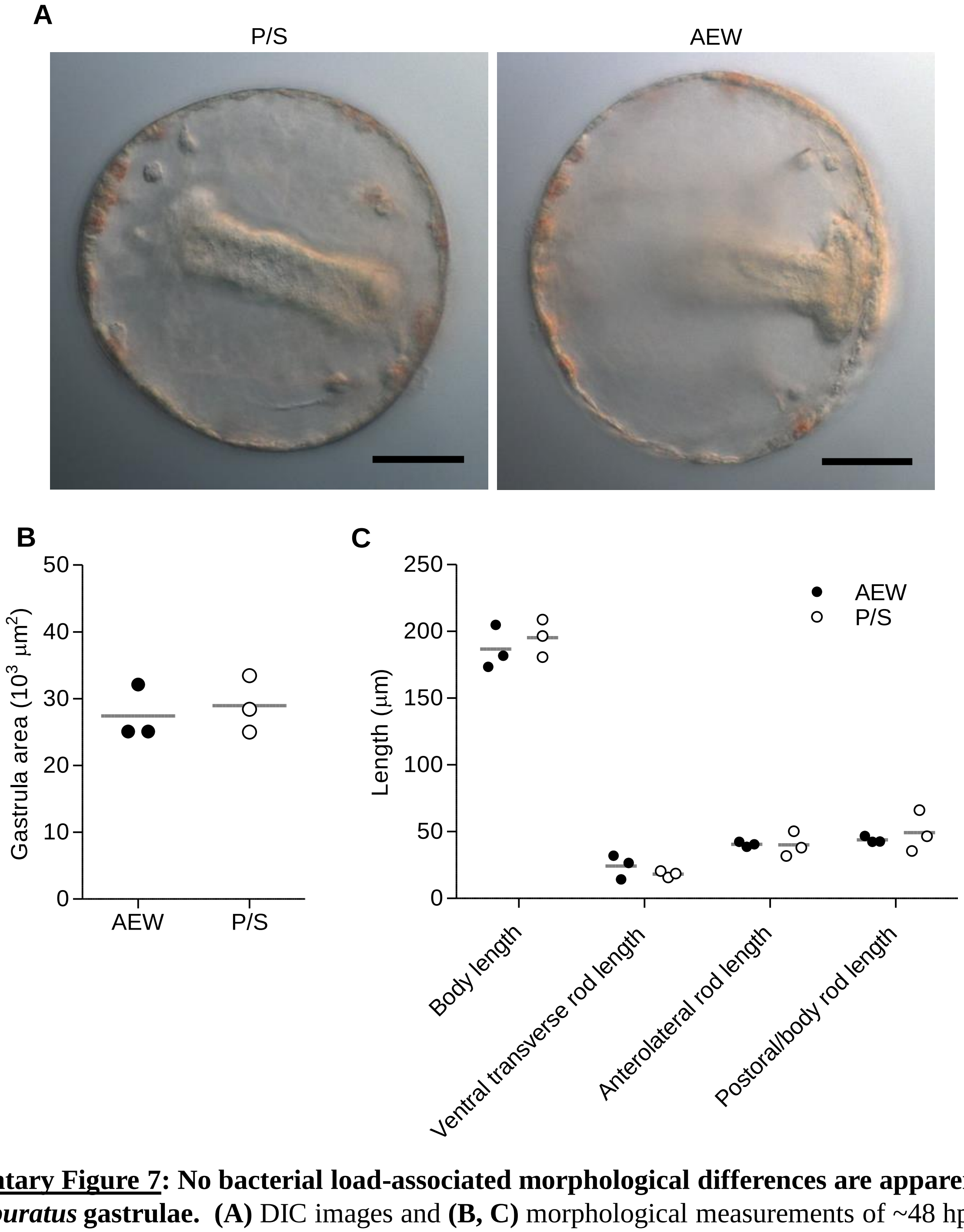

Supplementary Figure 7: No bacterial load-associated morphological differences are apparent in 48 hpf *S. purpuratus* gastrulae. (A) DIC images and **(B, C)** morphological measurements of ~48 hpf gastrulae raised in 0.2 μm-filtered FSW with antibiotics (P/S) or FSW with 20% 40 μm-filtered adult-exposed tank water (AEW). Scale bars = 50 μm.

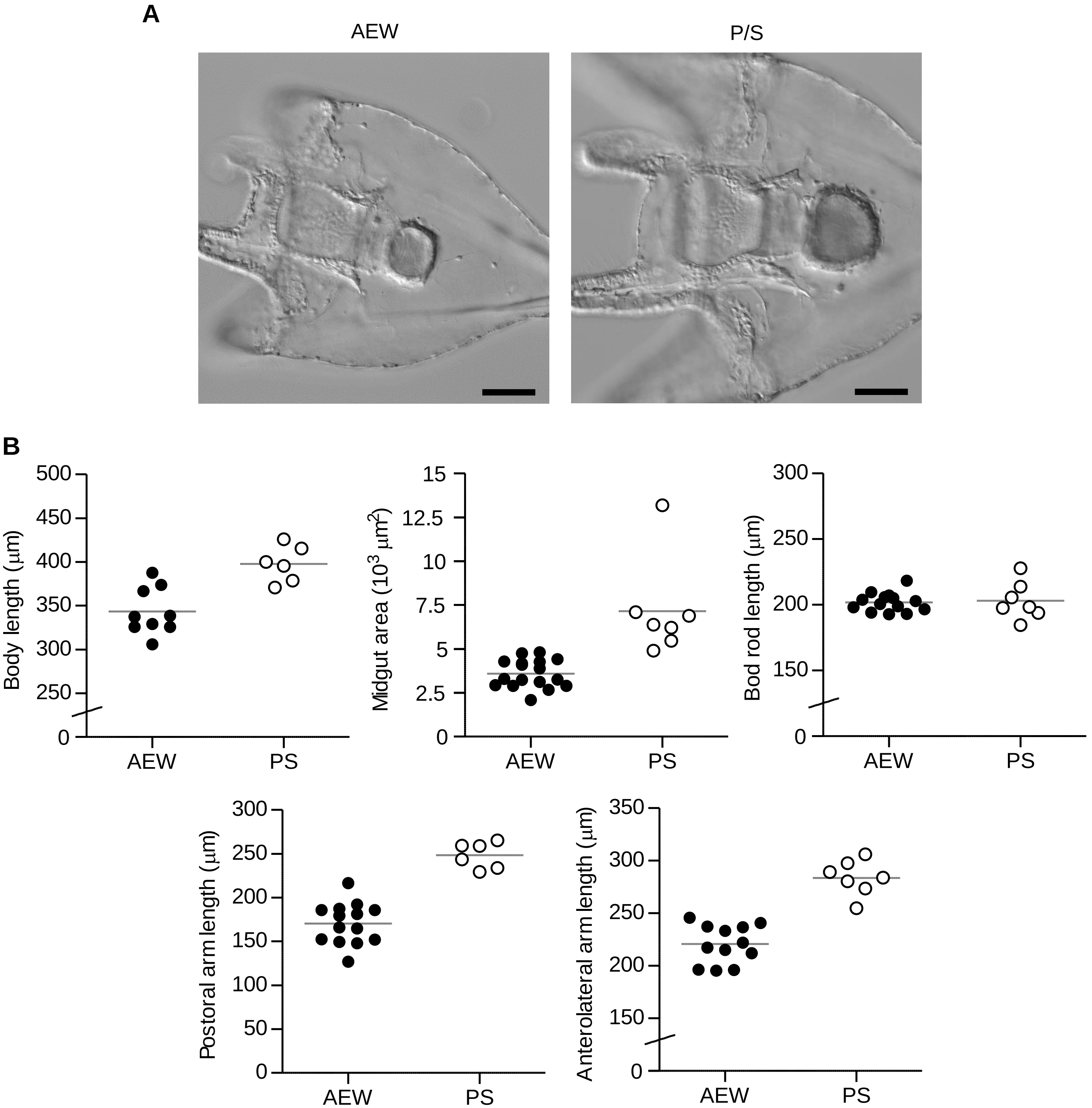

<u>Supplementary Figure 8</u>: **Bacterial load-induced developmental and morphological plasticity reproduced in an independent brood. (A)** DIC images and **(B)** morphological measurements of ~10 dpf pluteus larvae. Scale bars = 50 μm.

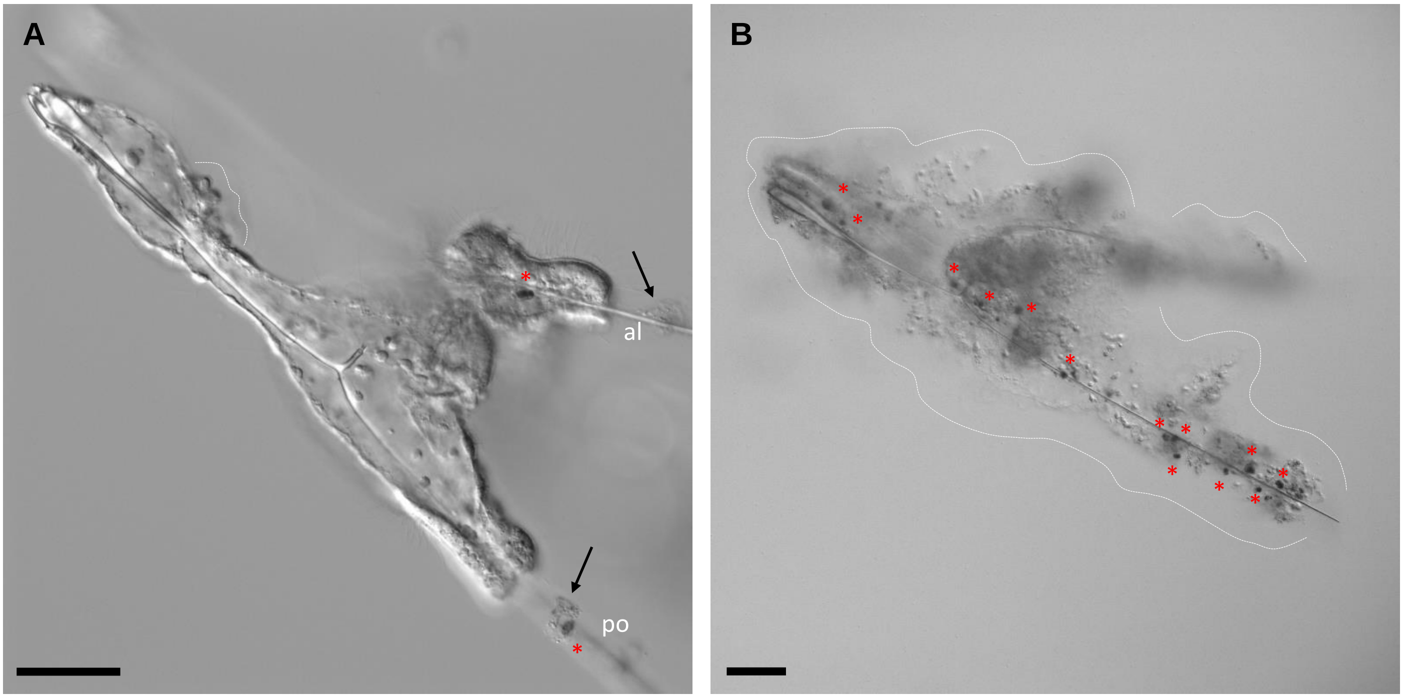

<u>Supplementary Figure 9</u>: Death from lethal *Vibrio lentus* infection. (A) Partially skeletonized larva. The postoral (po) and anterolateral (al) rods have punctured the ectoderm of the arm tips and are both associated with clumps of adherent *V. lentus* (black arrows). **(B)** Completely skeletonized larva. Dashed white lines indicate the margins of lysed larval contents and adherent *V. lentus*. Red asterisks indicate activated pigment cells. Scale bars = 50 μm.
